# Thymoquinone/β-*N*-acetylglucosaminidase, a novel plant-derived combination, inhibited quorum sensing signaling pathways and disrupted biofilm in *Staphylococcus aureus*

**DOI:** 10.3389/fcimb.2025.1686764

**Published:** 2025-10-23

**Authors:** Adel Attia M. Ahmad, El-Sayed Y. El-Naenaeey, Abeer S. Aloufi, Eman K. Khalifa, Tarek Khamis, Gamal A. Elmowalid, Marwa I. Abd El-Hamid

**Affiliations:** ^1^ Department of Microbiology, Faculty of Veterinary Medicine, Zagazig University, Zagazig, Egypt; ^2^ Department of Biology, College of Science, Princess Nourah bint Abdulrahman University, Riyadh, Saudi Arabia; ^3^ Department of Pharmacology, Faculty of Veterinary Medicine, Zagazig University, Zagazig, Egypt

**Keywords:** *Staphylococcus aureus*, thymoquinone, β-*N*-acetylglucosaminidase, biofilm, quorum sensing

## Abstract

**Background:**

Disrupting *Staphylococcus aureus S. aureus*biofilms is of utmost importance in the medical field. Identifying herbal compounds, especially those comprising enzymes, with antibacterial and biofilm-degrading properties offers a promising alternative to conventional biofilm inhibitors. The present study explored the novel effects of β-*N*-acetylglucosaminidase (NAGase), an enzyme derived from jack beans, in preventing biofilm formation and disrupting established biofilms. Furthermore, it investigated the potential effects of combining NAGase with thymoquinone (TQ) extracted from *Nigella sativa* seeds.

**Methods:**

*S. aureus* isolates with robust biofilm formation were selected using a quantitative microtiter plate method. The effect of TQ and/or NAGase on the growth and dispersion of existing biofilms was evaluated using a crystal violet staining assay in a microtiter plate. The study also investigated their impact on quorum sensing (QS) molecules (agrA, agrC, and sarA) and *icaA*, *agr*, and *atl* gene regulation using computational modeling and real-time PCR analysis, respectively.

**Results:**

Treatment with NAGase (2.5 U/mL) and TQ [1× minimum inhibitory concentration (MIC)] significantly reduced existing biofilms in multidrug-resistant and strong biofilm-forming *S. aureus* isolates by 40.9%–65.6% and 30.9%–69.3%, respectively. Notably, combining TQ and NAGase led to a greater reduction of established biofilm (61.8%–73.8%) compared to the untreated controls. Computer simulations revealed that the TQ ligand was a potent inhibitor of QS molecules, binding tightly to agrA and sarA. Notably, NAGase, whether used alone or in conjunction with TQ, led to a decrease in the expression of the *atl*, *icaA*, and *agr* genes.

**Conclusions:**

The combination of TQ and NAGase is a promising antibiofilm agent in *S. aureus*, offering several advantages over traditional options. It effectively targets both QS pathways and breaks down polysaccharide intercellular adhesins, in addition to exhibiting antibacterial properties to combat bacteria within existing biofilms. The presence of NAGase, a naturally occurring enzyme in cellular lysosomes, combined with the optimal pharmacokinetic properties of TQ, makes it a potential treatment for systemic and localized *S. aureus* infections.

## Introduction

Bacterial biofilms are fixed communities of bacteria encased in a protective matrix of polymeric extracellular polysaccharides. These biofilms contribute to antimicrobial resistance and impair the host’s immune defense system, thereby complicating the treatment of infections. Moreover, biofilm facilitates the colonization and survival of bacteria in adverse environmental conditions (Kluytmans and Wertheim, 2005). *Staphylococcus aureusS. aureus* produces extracellular polysaccharides (EPSs), which are mostly composed of polysaccharide intercellular adhesin (PIA) or poly-β-1-6-*N*-acetylglucosamine (PNAG) with multiple functions in pathogenesis, biofilm formation, and immune evasion (Maira-Litrán et al., 2002). The PIA accounts for approximately 90% of the overall biofilm composition, and it is composed of roughly 130 residues of *N*-acetylglucosamine (NAG or GlcNAc), with an estimated molecular weight of approximately 30 kDa (Ammendolia et al., 1999). Alpha-amylase is produced by *S. aureus*, where it hydrolyzes the 1,4-α-d-glycosidic bonds connecting glucose units. The sugars generated from this process are then transformed into glucose-6-phosphate, which plays an essential role in the synthesis of the exopolysaccharide matrix, PIA, found in biofilms (Lakshmi et al., 2013). The primary functions of PIA in the biofilm formation include the following: i) augmenting the hydrophobicity of the bacterial surface, which facilitates initial adhesion; ii) promoting the secondary accumulation of bacteria within the biofilm; and iii) creating a fibrous network that captures staphylococcal cells, thereby supporting biofilm growth and accumulation (Schommer et al., 2011). Moreover, the enduring presence of biofilms within catheters, even under mechanical stress, can primarily be attributed to the effective development of PIA (Chaieb et al., 2005). Four distinct genes, namely, *icaA*, *icaD*, *icaB*, and *icaC*, are organized within the icaADBC locus. The expression of these genes results in the production of fully functional PIA (Gerke et al., 1998). Notably, the expression of *ica* in *S. aureus* varies and is influenced by environmental factors, including glucose concentrations, the presence of anaerobic conditions, and exposure to antibiotics (Fluckiger et al., 2005).

In Gram-positive bacteria, peptidoglycan acts as a mechanical framework that preserves the shape of the cell. It is composed of linear chains of (1,4)-linked *N*-acetyl-d-glucosamine and acetylmuramic acid. Autolysin gene (*atl*) in *S. aureus* encodes a putative peptide consisting of two domains: *N*-acetylmuramyl-l-alanine amidase and a murein endopeptidase, which are classified as Glycosidic linkage–lytic enzyme (GL-lytic enzyme). These enzymes function as hydrolases for cell wall peptidoglycan, aiding in the initial adhesion of bacteria to polymer surfaces and promoting the early stages of biofilm formation, a process that can be triggered by acid stress or DNA damage (Neumann et al., 1993).

In the final stage of biofilm growth, biofilms may be dislodged by passive mechanisms such as shear stress or through active processes, which encompass enzymatic degradation, the action of antimicrobial agents, and the exhaustion of nutrients (Shen et al., 2018). Matrix-degrading enzyme (MDE) formulations offer a promising strategy to combat biofilms by breaking down their structural framework, hindering communication between cells, and precisely disrupting pre-existing biofilms. They comprise i) DNases, which facilitate the transition of sessile biofilm organisms to free-floating bacteria (Whitchurch et al., 2002); ii) combinations that incorporate the PIA-degrading enzyme Dispersin B, DNase I, and the cationic detergent cetylpyridinium chloride, which collaborate effectively to address biofilm formation (Izano et al., 2008; Kaplan et al., 2018); iii) co-administration of MDEs with chloroquine or ethidium bromide–DNA intercalators, which enhances their effectiveness (Chen et al., 2016b); iv) lipid-based nanoparticles encapsulating glycoside hydrolases and other microbial-derived enzymes (Hu et al., 2022); and v) MDE nanoformulations loaded with conventional antimicrobial agents (Tan et al., 2015). Specifically, the degrading enzyme *N*-acetylglucosaminidase (NAGase or GlcNAcase), belonging to the category of endo-*N*-acetyl-β-d-glucosaminidases (ENGases), hydrolyzes the glycosidic bond between an *N*-acetyl-β-d-glucosamine residue and the adjacent monosaccharide within an oligosaccharide chain, liberating NAG (Karamanos, 1997). Notably, NAGase is significantly utilized in the pharmaceutical sector (EFSA Panel on Food Contact Materials, Enzymes and Processing Aids (CEP), 2023) and acts as a marker for the deterioration of renal tubular cells, which is associated with kidney damage. Increased levels of NAGase are commonly found in individuals suffering from chronic kidney disease (Ali et al., 2014). Moreover, GlcNAcase is crucial for maintaining the precise balance of cellular *O*-linked GlcNAc levels (Zhang et al., 2018), and disturbances in this balance have been linked to type 2 diabetes, Alzheimer’s disease, and several types of cancer (Li et al., 2017). Biofilms, such as those created by PIA, possess a structural framework primarily composed of proteins, including amyloid proteins, peptidoglycans, and lipoteichoic acid. For example, certain mastitis isolates of *S. aureus* that are capable of biofilm formation can synthesize biofilm-associated protein (bap) within the mammary gland. These isolates demonstrate prompt adherence to non-living surfaces, facilitate intercellular adhesion, and contribute to the development of biofilms (Taglialegna et al., 2016). Serratiopeptidase, an enzyme with proteolytic activity sourced from *Serratia marcescens*, demonstrates antibacterial effects and diminishes the synthesis of pro-inflammatory substances such as prostaglandins and lipoteichoic acid, in addition to inhibiting biofilm development (Katsipis and Pantazaki, 2023). The quorum sensing (QS) system of *S. aureus* is controlled by the accessory global regulator (agr) cascades and the staphylococcal accessory regulator (sar) (Ganesh et al., 2022), which both collaborate to regulate the expression of biofilm encoding genes according to environmental conditions and population density and modulate common target genes following their attachment to identical DNA sequences (Archer et al., 2011).

Natural products have great potential as antimicrobials (Sieniawska and Georgiev, 2022) and have been thoroughly studied as significant sources of biocompatible antibiofilm compounds, as they are more effective and less hazardous than their synthetic counterparts (Sharifi et al., 2018; Zhao et al., 2018). Moreover, plant-derived components, i.e., baicalein (Chen et al., 2016a), quercetin (Liu et al., 2024), and emodin (Yan et al., 2017), are QS inhibitors. Thymoquinone (TQ) has potent antibacterial and antibiofilm properties, particularly against Gram-positive cocci like *S. aureus* and *Staphylococcus epidermidis* (Chaieb et al., 2011). It disrupts bacterial cell morphology and membrane integrity and inactivates biofilms (Fan et al., 2021). Furthermore, TQ enhances and restores the effectiveness of antibiotics against multidrug-resistant (MDR) *S. aureus* (Chaieb et al., 2011; Ahmad et al., 2024). Thymoquinone demonstrates protective effects against biofilm development and hemolytic activities, even at sub-minimum inhibitory concentrations, and reduces the expression of specific virulence factors (Tantivitayakul et al., 2020).

Regarding the aforementioned issues, this study is the first to investigate the application of β-NAGase derived from jack beans to disrupt biofilm formation, in addition to assessing the effectiveness of combining β-*N*-acetylglucosaminidase with TQ to disrupt established biofilms of *S. aureus*.

## Materials and methods

### Ethical approval

All procedures conducted in the current study received approval from the Faculty of Veterinary Medicine, Zagazig University, Egypt-Institutional Animal Care and Use Committee (VETCU-IACUC). All procedures strictly adhered to the guidelines and regulations and fully complied with the Animal Research Reporting of In Vivo Experiments guidelines (ARRIVE). Furthermore, for procedures involving human participants, written informed consent was obtained from all participants. Ethical approval for using human subjects was obtained from the designated health facility (National Research Centre, Giza, Egypt).

### Thymoquinone and β-*N*-acetylglucosaminidase

Thymoquinone (99%, 274666-1G), extracted from *Nigella sativa*, was purchased from Sigma Aldrich (St. Louis, MO, USA) and was prepared by dissolving 250 mg in 10% dimethyl sulfoxide. The resulting stock solution was stored at −20 °C. To obtain the required concentrations, 1 mL of the stock solution was diluted with an equal ratio of ethanol and water.

The NAGase enzyme, derived from the jack bean (*Canavalia ensiformis*), was sourced from Sigma Aldrich (St. Louis, MO, USA) with an activity of 5 units. It catalyzes the removal of *N*-acetylglucosamine residues from the non-reducing ends of glycoconjugates through hydrolysis.

### Bacterial isolates

Milk samples from 25 cows with chronic mastitis were collected from dairy farms in Zagazig, Egypt. Additionally, six pus, three urine, and two sputum samples from human subjects were gathered from a clinical bacteriology laboratory in February 2023. All samples were cultured on mannitol salt agar and incubated at 37°C for 24 h, the suspected colonies were cultured on sheep blood agar, and hemolytic colonies were further confirmed using Staph Latex Kits. *S. aureus* isolates were stored in brain heart infusion (BHI) broth with 30% glycerol at −20 °C for subsequent experiments.

### Disc diffusion assay

All confirmed *S. aureus* isolates were investigated for antimicrobial resistance patterns. Additionally, the susceptibility of *S. aureus* to TQ was examined using TQ-impregnated discs, as conducted according to the Clinical and Laboratory Standards Institute (CLSI) guidelines (Weinstein and Lewis, 2020). In brief, the antimicrobial discs of oxacillin (OX), cefepime (FF), clindamycin (DA), amoxicillin/clavulanic acid (AMC), trimethoprim/sulfamethoxazole (SXT), ciprofloxacin (CIP), chloramphenicol (C), doxycycline (DO), linezolid (LNZ), azithromycin (AZM), and gentamicin (CN), and TQ-impregnated discs (20 µL, 50 µg TQ) (Ahmad et al., 2024) were fixed onto Mueller–Hinton agar (MHA) plates previously inoculated with freshly recovered isolates with 0.5 McFarland standard turbidity. Plates were incubated at 37°C for 24 h, and inhibition zone sizes were recorded. The isolates displaying non-susceptibility to ≥3 different antimicrobial classes were categorized as MDR (Magiorakos et al., 2012).

### Broth microdilution technique

The recovered *S. aureus* isolates were tested for their susceptibility to TQ using a broth microdilution assay following CLSI guidelines (Weinstein and Lewis, 2020). To determine the minimum inhibitory concentration (MIC) of TQ, a serial dilution of TQ in 100 µL Mueller–Hinton broth (MHB) was prepared in a microtiter plate. A volume of 100 µL of a bacterial inoculum [10^6^ colony-forming units (CFU)/mL] was subsequently added to each well, and the plates were incubated at 37°C for 24 h. The lowest TQ concentration that inhibited visible bacterial growth was recorded as the TQ-MIC.

### Detection of biofilm formation

#### Congo red agar method

Freshly isolated *S. aureus* isolates were cultured on Congo red agar (CRA) plates. The medium was prepared by adding 800 mg of Congo red and 5% sucrose to 1 L of BHI agar (Oxoid, Basingstoke, Hampshire, UK). The inoculated plates were subsequently incubated at 37°C for 24 h. Isolates showing rough black or red smooth colonies were recorded as biofilm and non-biofilm producers, respectively (Freeman et al., 1989).

#### Tube method

Brain heart infusion broth was inoculated with a loopful of the tested *S. aureus* isolates from the overnight-incubated culture plates. The inoculated tubes were subsequently incubated at 37°C for 24 h. The tubes were decanted and washed with phosphate-buffered saline (PBS; pH 7.3). The dried tubes were stained with crystal violet (1%) for 10 min. The test tubes were then washed three times with sterile distilled water to remove the excess stain, and 5 mL of 33% (v/v) acetic acid was then added. The optical density (OD) at 600 nm (OD600) of crystal violet from each tube was measured using a microplate reader (Stat Fax 2100،, Palm City, FL, USA) (Christensen et al., 1985). Cut-off optical density value (OD cut = OD average of negative control + 3 × standard deviation of ODs of negative control) was used for categorizing *S. aureus* isolates based on biofilm-forming capacity, as follows: no biofilm producer (OD ≤ OD cut), weak biofilm producer (OD cut < OD ≤ 2 × OD cut), moderate biofilm producer (2 × OD cut < OD ≤ 4 × OD cut), and strong biofilm producer (OD > 4 × OD cut) (Shadkam et al., 2021).

#### Quantitative microtiter plate method

The biofilm-producing isolates in both tube and CRA methods were examined for quantitative biofilm production in microtiter plates. Biofilm biomass was quantified after staining with crystal violet as described elsewhere (Kim et al., 2022). A volume of 200 µL of BHI inoculated with 10^6^ CFU/mL of the tested isolates and 1% glucose was transferred to duplicate wells of a sterile 96-well flat-bottomed microtiter plate. Negative control wells with BHI only were included. Plates were covered and incubated aerobically at 37°C for 24 h, and then the wells were washed three times with 200 µL of physiological saline and dried to remove loosely adherent cells. Biofilms in each well were fixed with 200 μL of methanol. After 15 min, the plates were emptied and left to dry. The crystal violet binding assay was performed to quantify biofilm, and the interpretation of the results was described as previously detailed in the tube method section.

### Assessment of anti-biofilm activities of TQ, NAGase, and TQ/NAGase

#### Slime production inhibition

The antibiofilm activities of TQ, NAGase, and TQ/NAGase were evaluated, in triplicate, on CRA. A biofilm-producing strain was inoculated onto a CRA surface swabbed with 10 µL of TQ (1× MIC), NAGase (2.5 U/mL), or TQ/NAGase (1× MIC/2.5 U/mL). The plates were incubated at 37°C for 24 h, and the changes from black to red colonies were recorded (Lee et al., 2016).

#### Inhibition of biofilm production

To investigate the impact of TQ with varying concentrations (0.5–2× MIC), NAGase (2.5 U/mL), and TQ/NAGase (1× MIC/2.5 U/mL) on biofilm formation, the biofilm intensity of treated *S. aureus* isolates was measured and compared with that of the untreated ones. Briefly, 200 µL of BHI containing 10^6^ CFU/mL was distributed into a microtiter plate with evaluated antibiofilm agents. After aerobic incubation at 37°C for 24 h, the microtiter plate was washed, dried, and stained with 1% crystal violet for 10 min. Following destaining with 33% acetic acid, the OD600 of the crystal violet was measured using a microplate reader as previously described (Kim et al., 2022). The mean absorbance was used to calculate the percentage of inhibition of biomass formation using the following equation:

Percentage of inhibition = 100 − [(OD600 of treated isolates/OD600 of untreated ones) × 100] (Jadhav et al., 2013).

#### Eradication of established biofilm

The susceptibility of the existing biofilm to treatment with TQ, NAGase, and their combination was analyzed. The effectiveness of these treatments was evaluated by comparing CFU counts and the OD600 readings of crystal violet-stained biofilms to those of the untreated controls as previously conducted (Fan et al., 2021; Kim et al., 2022). In brief, biofilm cultivation was performed by inoculating 200 µL of BHI broth containing 10^6^ CFU/mL of bacterial inoculum in microtiter plate wells. After incubating at 37°C for 24 h, the BHI broth was discarded, and each well was washed with PBS. Subsequently, 200 µL of different treatments was added: 0.5× and 1× MIC of TQ, NAGase (2.5 U/mL), and a combination of TQ/NAGase (1× MIC/2.5 U/mL). The plates were incubated for another 24 h at 37°C. To measure the number of viable bacteria, the tested inocula were mixed, serially diluted in sterile PBS, and expressed as log_10_ CFU/mL. The biofilm quantification and percentages of biofilm reduction following different treatments were assessed employing the methodology and formula described earlier.

#### Quantitative real-time PCR

To understand the genetic regulation of biofilm production, autolysin, and QS systems in strong biofilm-producing and MDR *S. aureus* isolates, quantitative real-time PCR (qRT-PCR) was employed to analyze the expression of *icaA*, *atl*, and *agr* genes, respectively, on biofilm cultures treated with NAGase (2.5 U/mL) and TQ/NAGase (1× MIC/2.5 U/mL), as well as untreated controls. Total RNA was extracted using Qiagen’s RNeasy mini kit (Qiagen, Hilden, Germany) following the manufacturer’s instructions. The RNA concentration was then measured using a QuaWell UV-VIS spectrophotometer (Quawell Technology, Inc., San Jose, California, USA). The purity of the isolated mRNA was assessed by measuring the absorbance ratio (A260/A280). The relative expression of the analyzed genes was determined using the QuantiTect SYBR Green one-step RT-PCR kit (Qiagen, Germany) as specified by the manufacturer and Mx3000P real-time qPCR equipment (Stratagene, La Jolla, CA, USA). The *16S rRNA* gene, as a housekeeping gene, was stably expressed across treatments to optimize the data, as checked using GeNorm. The sequences of the primers targeting *icaA*, *atl*, *agr*, and *16S rRNA* genes were F: TCTCTTGCAGGAGCAATCAA and R: TCAGGCACTAACATCCAGCA, F: ACACCACGATTAGCAGAC and R: AGCTCCGACAGATTACTT, F: GTGCCATGGGAAATCACTCCTTCC, R: TGGTACCTCAACTTCATCCATTATG, and F: GGACTGTTATATGGCCTTTT and R: GAGCCGTTCTTATGGACCT, respectively (Miao et al., 2019).

The thermal cycling program was initial denaturation at 94°C for 5 min, then 30 cycles of denaturation at 94°C for 1 min, annealing at primer melting temperature for 1 min, and extension at 72°C for 2 min with a final extension step at 72°C for 7 min. The relative expression of the investigated genes was determined using the ΔΔCt method (Schmittgen and Livak, 2008).

#### *In silico* molecular docking

The interactions between TQ (CID: 10281) and several QS molecules—agrA (3BS1), agrC (4BXI), and sarA (2FNP)—were analyzed using *in silico* methods. The AutoDock MGL tools were employed for molecular preparation and grid box setup, while the AutoDock Vina and BIOVIA Discovery Visualization software were used for docking simulations (Trott and Olson, 2010). The preliminary evaluation of docking results was performed using AutoDock Vina, focusing on binding affinity and root mean square deviation (RMSD) values. RMSD was used to assess ligand pose deviation across docking modes. Although full molecular dynamics (MD) simulations were not conducted, these parameters provided initial insights into binding stability and pose reliability (Trott and Olson, 2010).

### Statistical analysis

The data were analyzed using SPSS version 26 (IBM Corp., Armonk, NY, USA). The chi-square test was used to assess the differences in the antimicrobial resistance patterns and biofilm production abilities of the recovered isolates from various sources. For the evaluation of different biofilm methods, the concordance between the test tube and microtiter plate was assessed using kappa (κ) statistics. An independent sample t-test was used to determine the difference between the biofilm production of *S. aureus* isolates from different sources using microtiter plate and test tube methods. The visualization of the prevalence of resistance to various antimicrobial agents and differences in biofilm production abilities of *S. aureus* isolates was conducted using stacked bar plots, where subcolumns are part of the total column, using the ggplot (Wickham et al., 2007) package in the R software version 4.3.3 (https://www.r-project.org/). Additionally, one-way ANOVA and Tukey’s *post-hoc* test were used to evaluate the antibiofilm efficacy of TQ (at various concentrations), NAGase, and their combination against MDR *S. aureus* isolates. The normality and homogeneity among the treatment groups were determined utilizing the Shapiro–Wilk and Levene tests, respectively. All experimental procedures were conducted in triplicate, and the results were expressed as mean ± standard error of the mean (SEM). The *p*-values were considered statistically significant if they were less than 0.05. Graphs were generated using GraphPad Prism version 8 (San Diego, CA, USA) and R-software version 4.4.3 (Team, 2015) using ggplot (Wickham et al., 2007), pheatmap (Kolde, 2019), and factoextra (Kassambara and Mundt, 2016) packages.

## Results

### Susceptibility of *S. aureus* to antimicrobials and TQ on agar plates

**Figure 1:**
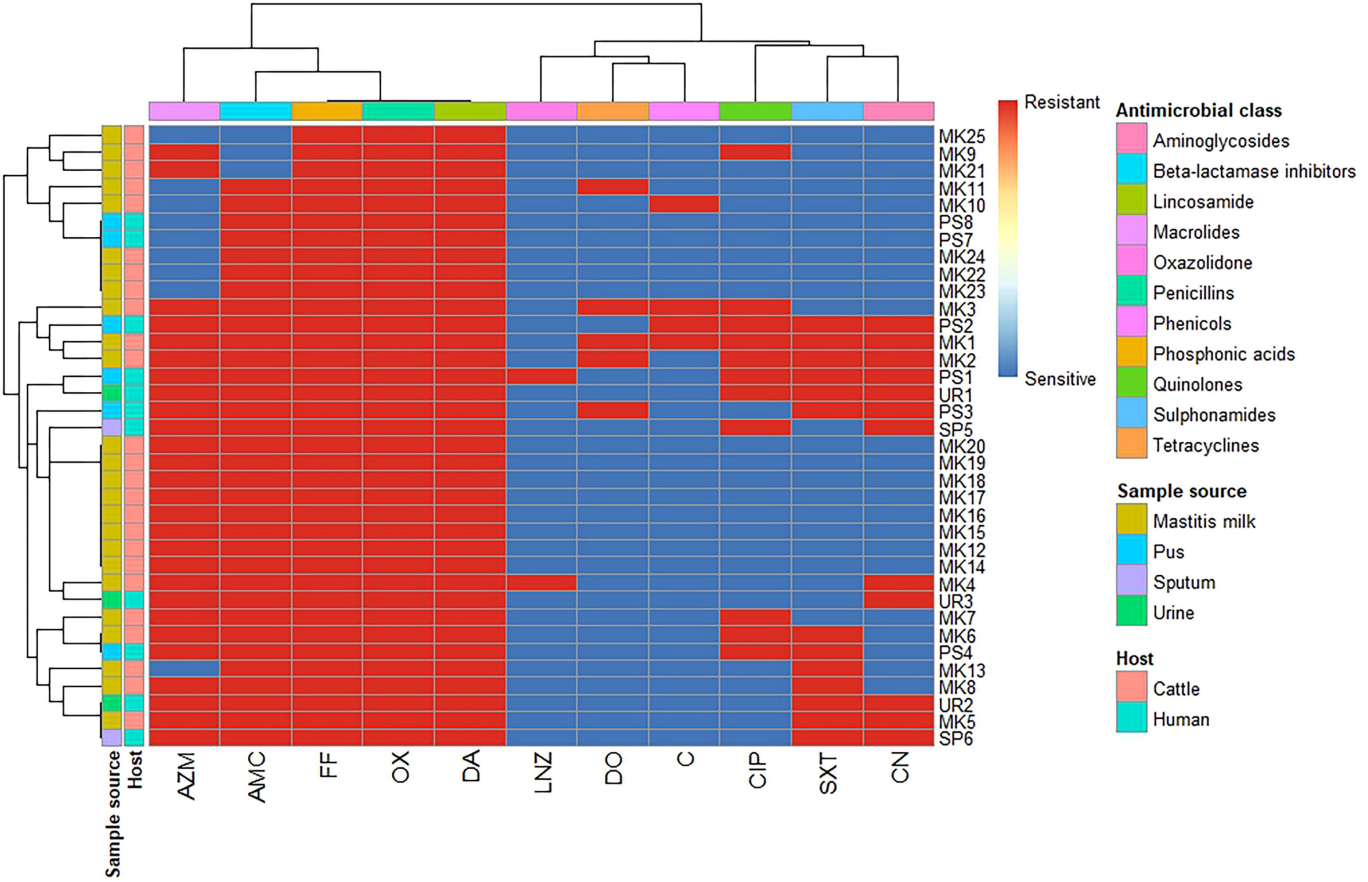
Hierarchical clustering heatmap showing the overall distribution of the investigated *Staphylococcus aureus* isolates based on the phenotypic antimicrobial resistance pattern. MK, milk; PS, pus; SP, sputum; UR, urine; OX, oxacillin; DA, clindamycin; FF, fosfomycin; AMC, amoxicillin/clavulanic acid; AZM, azithromycin; SXT, trimethoprim/sulfamethoxazole; CIP, ciprofloxacin; DO, doxycycline; CN, gentamycin; C, chloramphenicol; LNZ, linezolid.

**Figure 2:**
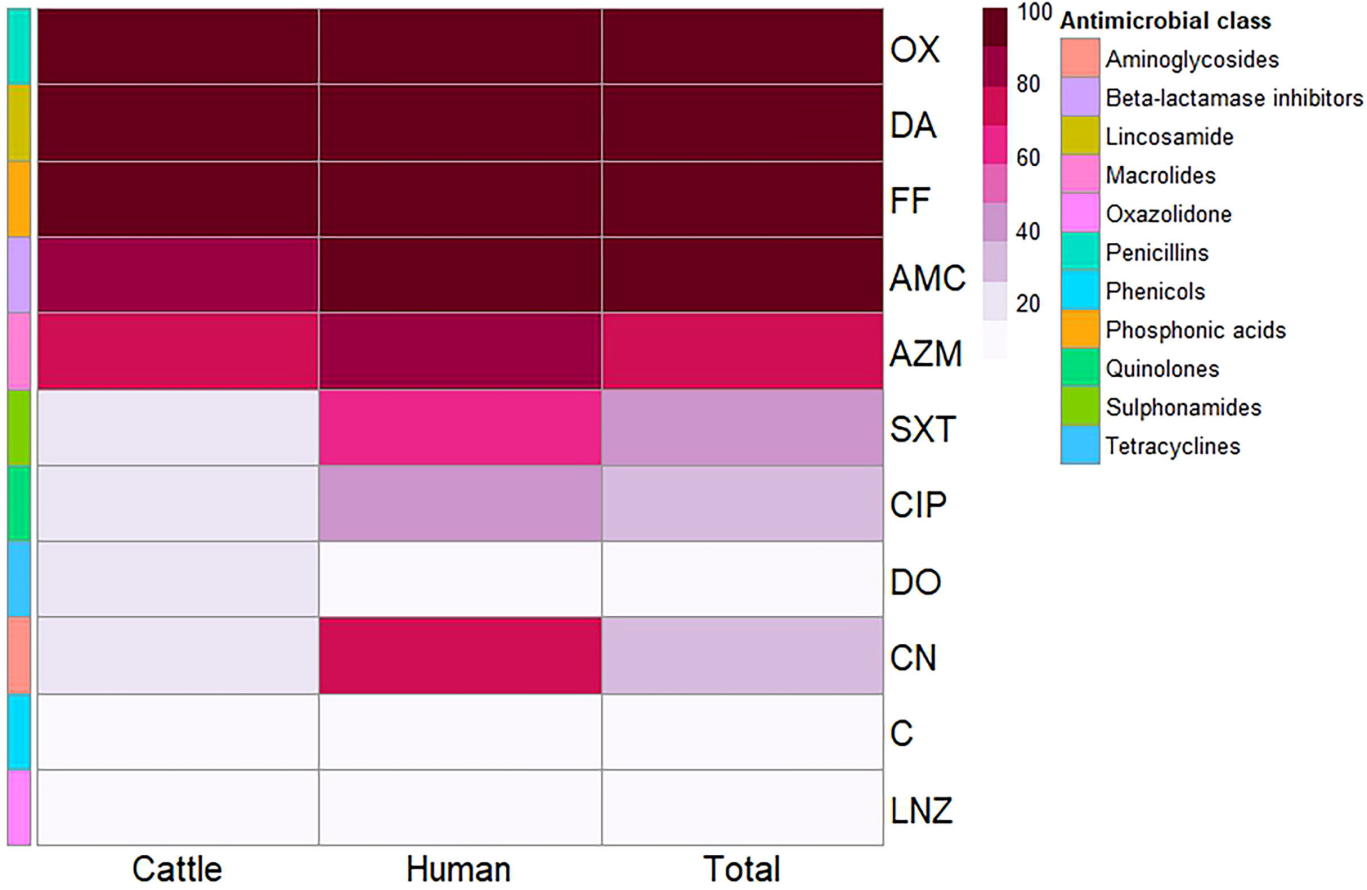
Heatmap showing the antimicrobial resistance patterns of the investigated *Staphylococcus aureus* isolates from different sources. Color code on the right of the heatmap refers to the percentage of resistance to the investigated antimicrobial agent. Different antimicrobial classes are color-coded on the right of the heatmap. OX, oxacillin; DA, clindamycin; FF, fosfomycin; AMC, amoxicillin/clavulanic acid; AZM, azithromycin; SXT, trimethoprim/sulfamethoxazole; CIP, ciprofloxacin; DO, doxycycline; CN, gentamycin; C, chloramphenicol; LNZ, linezolid.

A total of 36 *S. aureus* isolates were tested for antimicrobial susceptibility using disc diffusion (**Figure 1**, **Supplementary Tables S1**, **S2**). The 25 isolates obtained from mastitis milk demonstrated the following characteristics: i) a total of 16 distinct antimicrobial susceptibility patterns; ii) resistance to 3–10 of the 11 tested antimicrobials; iii) complete resistance to oxacillin, fosfomycin, and clindamycin; and iv) notable sensitivity to linezolid, chloramphenicol, gentamicin, and doxycycline (96%, 88%, 84%, and 84%, respectively). The 11 isolates derived from human samples demonstrated the following characteristics: i) 9 distinct antimicrobial susceptibility patterns; ii) resistance to 4–9 of the 11 antimicrobials tested; iii) complete resistance to oxacillin, amoxicillin/clavulanic acid, fosfomycin, and clindamycin; and iv) remarkable sensitivity to linezolid, doxycycline, and chloramphenicol (90.9% each). The isolates from human sources exhibited higher resistance rates to antimicrobial agents, along with a wider range of unpredictable resistance patterns. Statistical analysis demonstrated significant differences in the antimicrobial resistance patterns of the tested *S. aureus* isolates obtained from different sources to trimethoprim/sulfamethoxazole and gentamycin antimicrobials (*p* = 0.032 and 0.002, respectively) (**Figure 2**).

**Figure 3:**
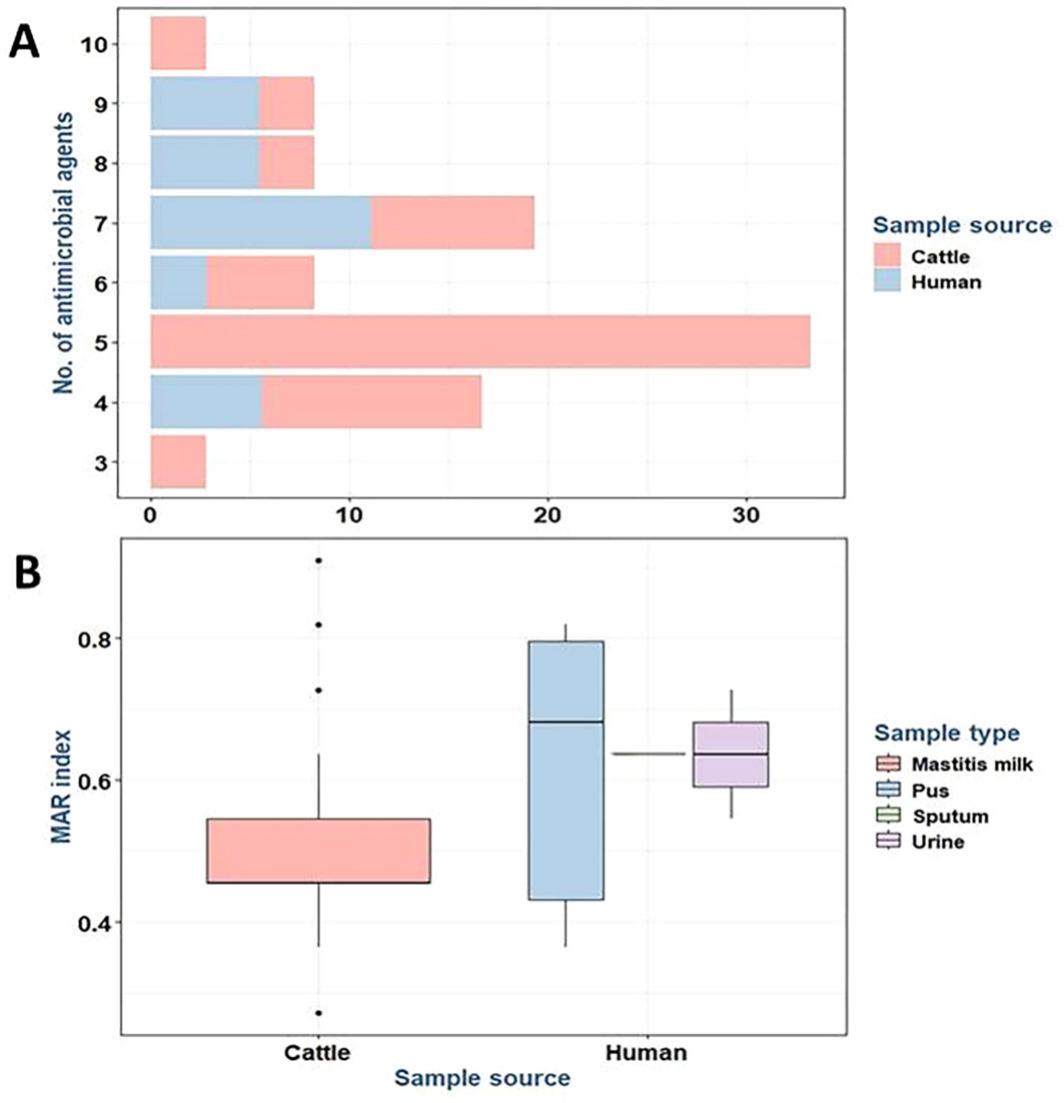
Stacked bar plot showing the resistance to various antimicrobial agents in *Staphylococcus aureus* isolates from different sources **(A)**. Box plot showing the MAR index of the tested *S. aureus* isolates from different sources **(B)**. MAR, multiple antibiotic resistance.

In addition, all *S. aureus* isolates were categorized as MDR exhibiting multiple antibiotic resistance (MAR) indices ranging from 0.27 to 0.91. Furthermore, there were statistically significant differences in the prevalence of resistance to five antimicrobial agents and classes with a MAR index of 0.45 (*p* = 0.006) among *S. aureus* isolates from different sources (**Figure 3**).

### Antimicrobial activity of thymoquinone

Thymoquinone antimicrobial activity was assessed on a total of 16 MDR *S. aureus* isolates exhibiting resistance to multiple antimicrobials (**Table 1**). The minimum concentration of TQ necessary to inhibit growth ranged from 2 to 100 µg/mL. Nevertheless, the results from the disc diffusion test did not align with the MIC determined via broth microdilution assay.

**Table 1:** Antimicrobial activity of thymoquinone against 16 multidrug-resistant *StaphylococcusS. aureus* isolates along with their biofilm-forming ability.

Isolate code no.	Antimicrobial activity of TQ		Biofilm formation		
	IZD (mm)	MIC (µg/mL)	Colony color on CRA	OD600 (biofilm intensity)	
				Test tube	Microtiter plate
MK1	18	5	Black	0.230 (WA)	0.617 (SA)
MK2	11	20	Red	0.160 (WA)	0.221 (WA)
MK3	16	50		0.150 (WA)	0.199 (WA)
MK4	19	5		0.155 (WA)	0.210 (WA)
MK6	17	3	Black	0.248 (MA)	0.676 (SA)
MK7	12	100		0.293 (MA)	0.398 (MA)
MK8	15	10		0.342 (MA)	0.454 (MA)
PS1	13	20		0.368 (SA)	0.874 (SA)
PS2	19	5		0.485 (SA)	0.901 (SA)
PS3	18	5		0.547 (SA)	0.833 (SA)
SP6	19	3		0.522 (SA)	0.620 (SA)
UR1	18	3		0.384 (SA)	0.726 (SA)
UR2	17	5		0.635 (SA)	0.553 (SA)
UR3	20	3		0.440 (SA)	0.535 (SA)
PS4	21	2		0.240 (MA)	0.673 (SA)
SP5	20	3		0.588 (SA)	0.605 (SA)

MK, milk; PS, pus; SP, sputum; UR, urine; TQ, thymoquinone; IZD, inhibition zone diameter; MIC, minimum inhibitory concentration; CRA, Congo red agar; OD, optical density; WA, weakly adherent; MA, moderately adherent; SA, strongly adherent.

### Biofilm formation capacity on Congo red agar plates

A total of 16 isolates of *S. aureus* were analyzed to identify biofilm production. The appearance of black colonies on Congo red agar plates indicated successful biofilm formation (**Table 1**). Notably, isolates derived from mastitis milk samples exhibited black or red colonies, whereas those obtained from human sources consistently yielded black colonies.

### Variability of biofilm formation via test tube and microtiter plate methods

The capacity for biofilm formation among 16 MDR *S. aureus* isolates was assessed through OD measurements at 600 nm (OD600). The findings indicated a range of biofilm adherence levels across the isolates (**Table 1**). In the test tube method, isolates derived from mastitis milk samples demonstrated weak-to-moderate biofilm formation with OD600 values ranging from 0.150 to 0.342. In contrast, the majority of isolates obtained from human sources exhibited strong biofilm-forming phenotypes with OD600 values between 0.368 and 0.635. Using the microtiter plate method, isolates obtained from mastitis milk samples showed weak, moderate, and strong biofilm adherence, with OD600 values varying from 0.199 to 0.676. In comparison, all isolates sourced from humans displayed strong biofilm adherence, with OD600 values ranging from 0.535 to 0.901. The microtiter plate method proved to be the most accurate, sensitive, and dependable screening technique for identifying the production of biofilms by staphylococci.

**Figure 4:**
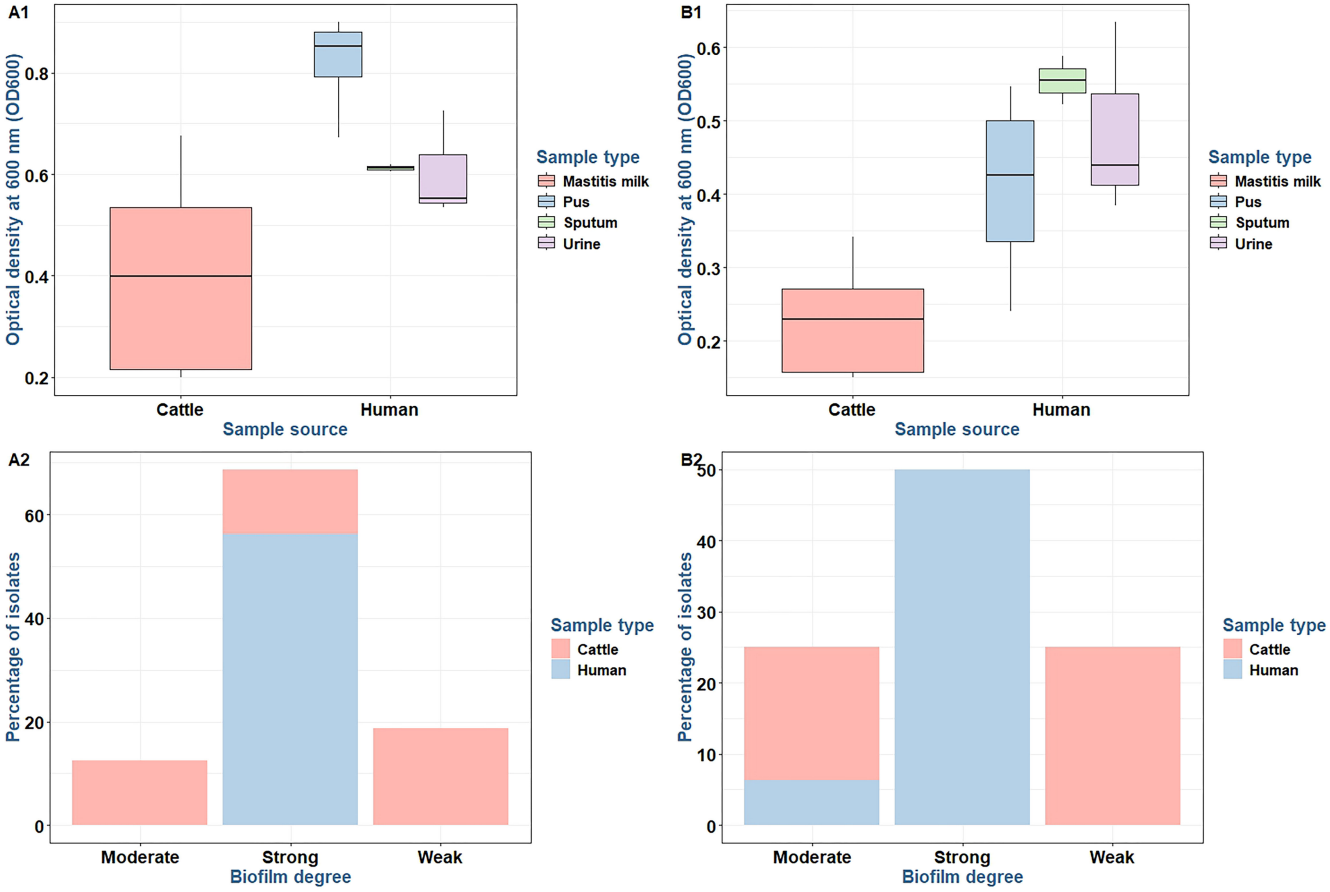
Biofilm production **(A1, B1)** and differences in biofilm production abilities **(A2, B2)** between *Staphylococcus aureus* isolates using microtiter plate **(A1, A2)** and test tube **(B1, B2)** methods.

There were statistically significant differences in the biofilm production abilities of *S. aureus* isolates obtained from various sources by microtiter plate (*p* = 0.003) and test tube (*p* < 0.0001) methods. Among strong biofilm degrees by the microtiter plate method, there were statistically significant differences (*p* = 0.005) between *S. aureus* isolates from various sources. Among weak and strong biofilm degrees by the test tube method, there were statistically significant differences (*p* = 0.019 and 0.001, respectively) between *S. aureus* isolates from various sources (**Figure 4**). The concordance between microtiter plate and test tube methods was moderate for *S. aureus* isolates (kappa value: κ = 0.676, 95% CI = 0.357–0.995) (**Table 2**).

**Table 2:** Comparative evaluation of biofilm formation in *Staphylococcus aureus* isolates using microtiter plate and test tube assays.

Test	No. of isolates (%)			Concordance (k), (95% CI)	*p*-Value
	Microtiter plate				
	WA	MA	SA		
Test tube					
WA	3 (18.8)	0	1 (6.3)	0.676 (0.357–0.995)	<0.0001
MA	0	2 (12.5)	2 (12.5)		
SA	0	0	8 (50)		

WA, weakly adherent; MA, moderately adherent; SA, strongly adherent; CI, confidence interval.

### Impact of TQ and/or NAGase in biofilm formation

**Figure 5:**
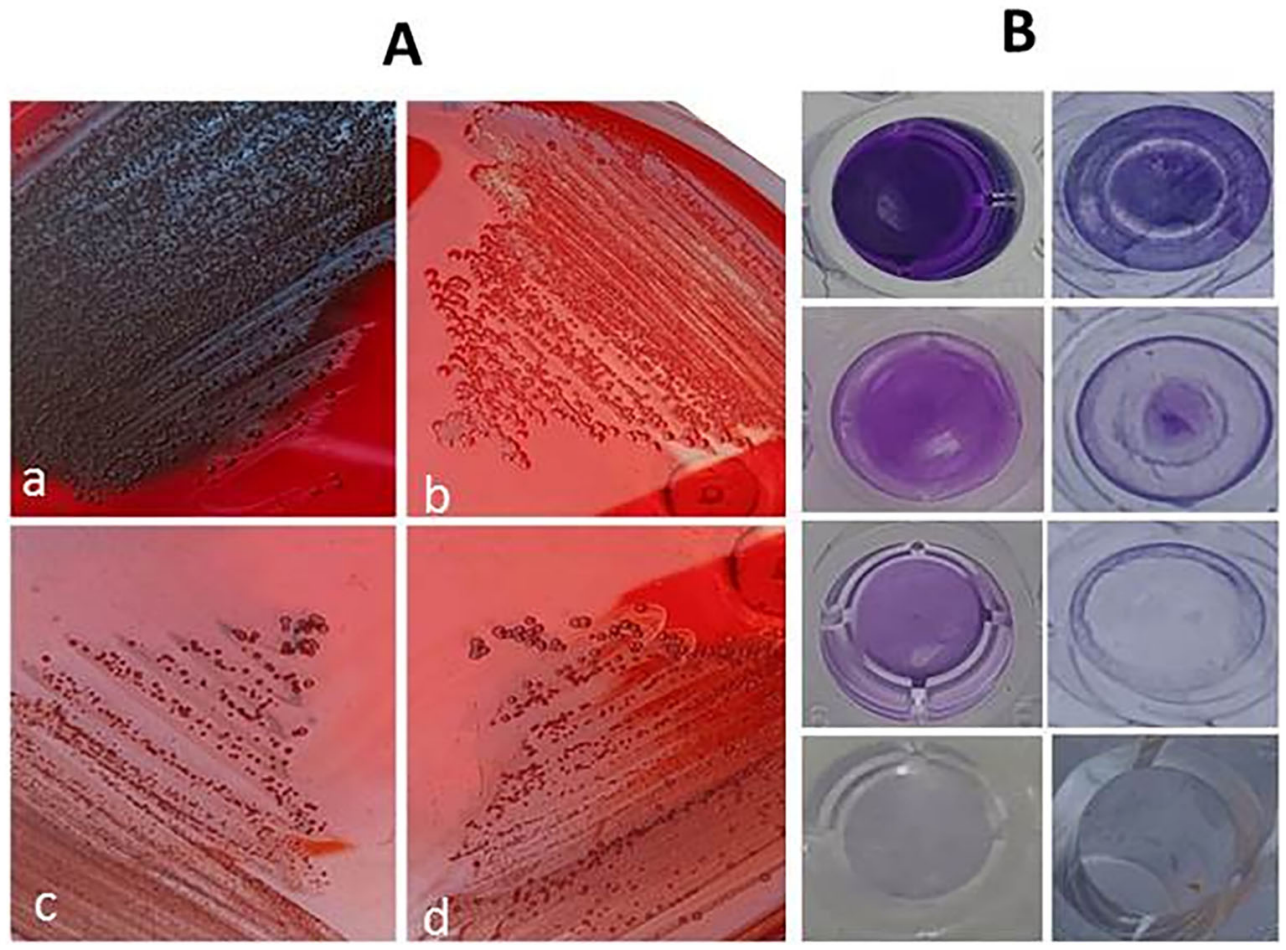
Inhibition of biofilm formation on Congo red agar medium **(A)** showing color alterations from black to red; black colonies of untreated biofilm producer **(a)**, red colonies of non-biofilm producer **(b)**, red colonies of thymoquinone-treated isolate **(c)**, and red colonies of thymoquinone/β-*N*-acetylglucosaminidase-treated isolate **(d)**. Eradication of established biofilm on microtiter plate **(B)** showing strong biofilm producer, biofilm eradication by N-acetylglucosaminidase, significant biofilm eradication by thymoquinone/β-N-acetylglucosaminidase, and broth control from top to bottom.

A selection of 10 *S. aureus* isolates (MK1, MK6, UR1, UR2, UR3, PS1, PS2, PS3, SP5, and SP6) was made based on their robust biofilm formation capabilities and MDR patterns as detailed in **Table 3**. Notably, the majority of these isolates exhibited sensitivity to TQ, with MICs ranging from 3 to 20 µg/mL (**Table 1**). The application of 1× MIC of TQ and combination of TQ with NAGase (1× MIC/2.5U/mL) to the surface of the Congo red medium inhibited biofilm formation, leading to the development of red colonies (**Figure 5A**). Moreover, treatment with TQ at concentrations between 0.5 and 2 times the MIC resulted in a significant decrease in biofilm formation among the isolates (**Table 3**). Specifically, TQ led to a reduction in the biofilm formation of 40.5%–57.9% at 0.5× MIC, 51.2%–77.4% at 1× MIC, and 59.4%–83.9% at 2× MIC after 24 h of exposure. The study also examined the effects of NAGase (2.5 U/mL) and/or TQ at 1× MIC on biofilm reduction. The findings indicated that NAGase alone (2.5 U/mL) decreased biofilm formation by 38.6%–58.4%. Furthermore, the combination of TQ with NAGase (1× MIC/2.5 U/mL) resulted in an even more significant reduction in biofilm, ranging from 64% to 82.4%.

**Table 3:** Inhibition of biofilm formation of *StaphylococcusS. aureus* isolates treated with thymoquinone and/or β-*N*-acetylglucosaminidase.

Isolate code no.	Percentage of mean OD600 reduction of treated isolates				
	TQ MIC (μg/mL)			NAGase (2.5 U/mL)	TQ/NAGase (1× MIC/2.5 U/mL)
	2×	1×	0.5×		
MK1	72.0	67.4	50.8	38.6	73.5
MK6	83.9	77.4	47.3	43.2	80.7
UR1	74.0	70.4	53.2	41.5	66.4
UR2	70.2	61.8	40.5	54.9	82.4
UR3	59.4	57.0	42.1	50.3	64.0
PS1	81.1	76.1	48.1	49.1	73.6
PS2	76.2	67.0	52.6	47.0	79.7
PS3	74.4	62.1	57.9	40.3	76.1
SP5	66.7	51.2	49.2	48.6	77.7
SP6	69.6	63.2	52.6	58.4	73.4

MK, milk; UR, urine; PS, pus; SP, sputum; OD, optical density; TQ, thymoquinone; MIC, minimum inhibitory concentration; NAGase, β-*N*-acetylglucosaminidase.

**Figure 6:**
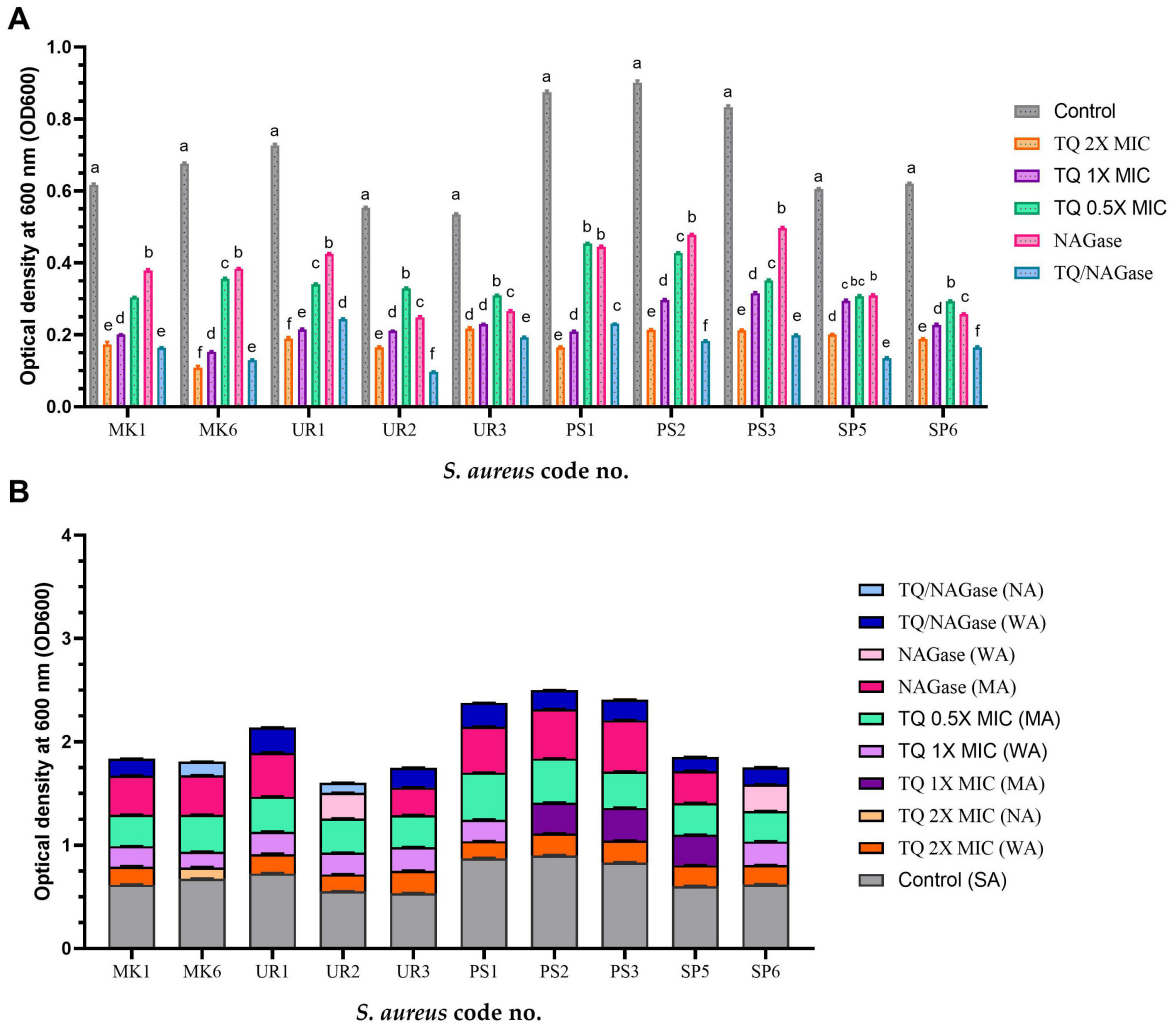
Inhibition of biofilm formation of *StaphylococcusS. aureus* isolates treated with thymoquinone (TQ) and/or β-*N*-acetylglucosaminidase (NAGase) **(A)** and differences in biofilm production abilities among control untreated *S. aureus* isolates and TQ and/or NAGase-treated isolates **(B)**. Results were expressed as the mean of three independent experiments ± standard error of the mean (SEM). **(a–f)** Means with various superscript letters vary significantly at *p* < 0.0001. MK, milk; UR, urine; PS, pus; SP, sputum; OD, optical density; TQ, thymoquinone; MIC, minimum inhibitory concentration; NAGase, β-*N*-acetylglucosaminidase; NA, non-adherent; WA, weakly adherent; MA, moderately adherent; SA, strongly adherent.

There were significant differences in the biofilm inhibition among various concentrations of TQ, NAGase, and their combination against 10 MDR *S. aureus* isolates (*p* < 0.0001). TQ/NAGase showed the most significant (*p* < 0.0001) reduction in biofilm formation in seven treated isolates, when compared with the control untreated ones. TQ 2× MIC showed the most significant (*p* < 0.0001) reduction in biofilm formation in three isolates with code nos. PS1, UR1, and MK6, when compared with the control untreated ones (**Figure 6A**).

The combination of TQ and NAGase treatment resulted in a marked reduction of adherence from strong to weak in all tested isolates, with the exception of two isolates with code nos. MK6 and UR2, which exhibited complete inhibition and became non-adherent (**Figure 6B**).

### Effects of TQ and/or NAGase on the established biofilm

**Table 4**, **Figure 5B** display the effects of TQ and/or NAGase on the existing biofilm of *S. aureus* isolates. The results showed that TQ significantly reduced the established biofilm by 22.5%–58.5% at 0.5× MIC and 30.9%–69.3% at 1× MIC after 24 h of incubation at 37°C. Although 1× MIC of TO treatment effectively broke down biofilms, resulting in the release of bacteria, it ultimately led to a decrease in the viable CFUs, acting as a bactericidal agent. A notable reduction of existing biofilm was recorded after treatment with NAGase (2.5 U/mL), ranging from 40.9% to 65.6%. Notably, the combination of TQ and NAGase demonstrated the highest effectiveness in eliminating established biofilms, achieving a reduction percentage between 61.8% and 73.8% when compared to that in the untreated biofilm.

**Table 4:** Established biofilm inhibition of *StaphylococcusS. aureus* isolates treated with thymoquinone and/or β-*N*-acetylglucosaminidase.

Isolate code no.	Percentage of mean OD600 reduction/log_10_ CFU of treated isolates			
	TQ MIC (μg/mL)		NAGase (2.5 U/mL)	TQ/NAGase (1× MIC/2.5 U/mL)
	0.5×	1×		
MK1	30.1/7.77	38.0/7.30	44.1/7.89	66.5/7.94
MK6	45.0/7.92	66.5/7.63	54.0/7.99	70.3/8.09
UR1	28.5/7.83	37.6/7.39	49.0/7.32	71.0/7.45
UR2	23.1/7.59	53.7/7.0	50.6//7.96	73.7/8.11
UR3	58.5/7.54	62.4/7.87	53.3/7.97	62.5/8.04
PS1	56.2/8.1	68.0/7.66	57.7/7.95	64.2/8.7
PS2	31.5/7.53	45.5/8.91	64.9/8.06	72.5/8.10
PS3	41.1/7.85	69.3/7.50	65.6/8.09	73.8/8.11
SP5	39.6/7.86	48.2/7.46	40.9/7.86	61.8/8.04
SP6	22.5/7.61	30.9/7.32	53.2/7.81	69.1/8.2

MK, milk; UR, urine; PS, pus; SP, sputum; OD, optical density; TQ, thymoquinone; MIC, minimum inhibitory concentration; NAGase, β-*N*-acetylglucosaminidase; CFU, colony-forming units.

**Figure 7:**
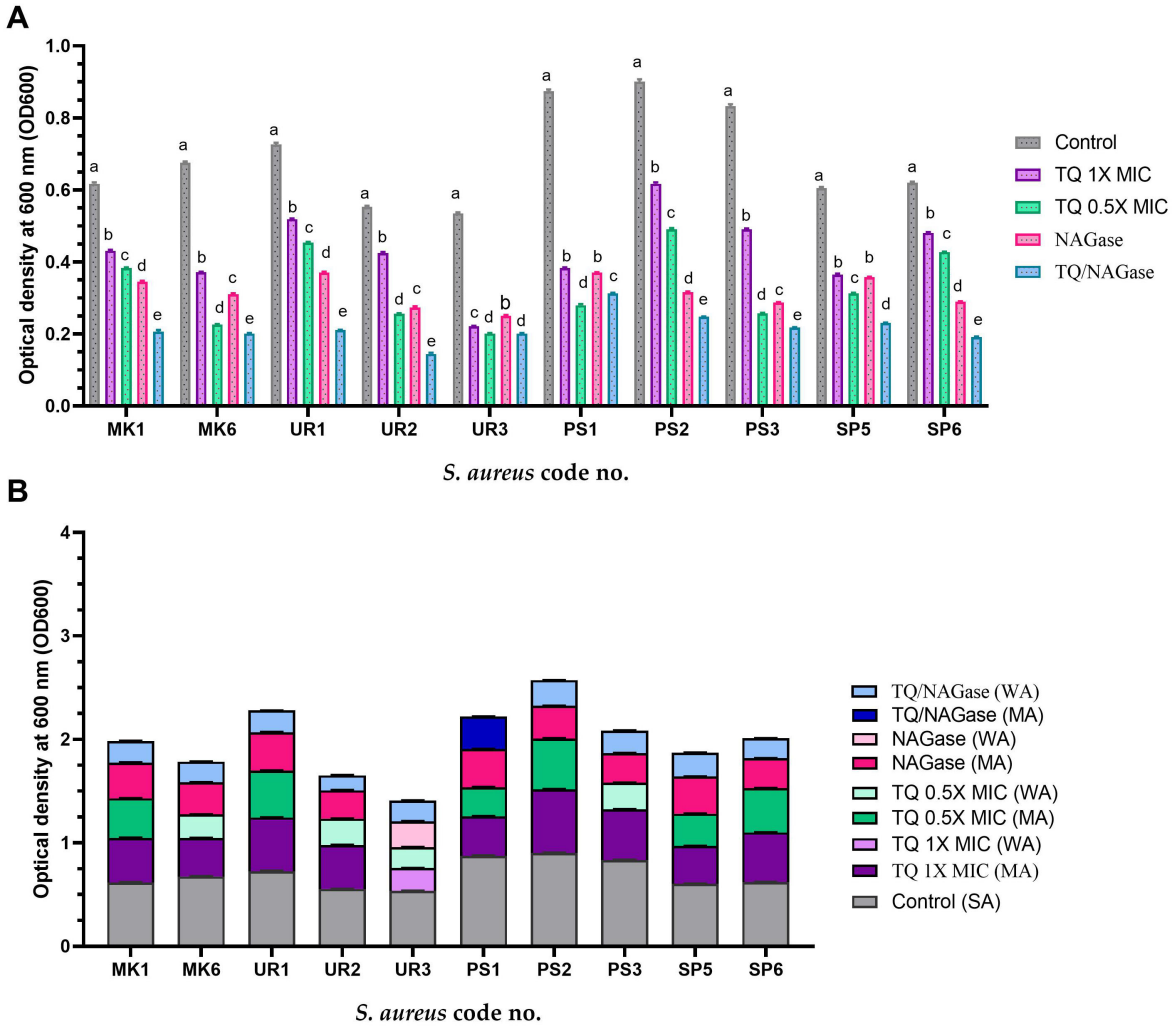
Established biofilm inhibition of *StaphylococcusS. aureus* isolates treated with thymoquinone (TQ) and/or β-*N*-acetylglucosaminidase (NAGase) **(A)** and differences in biofilm production abilities among control untreated *S. aureus* isolates and TQ and/or NAGase-treated isolates **(B)**. Results were expressed as the mean of three independent experiments ± standard error of the mean (SEM). **(a–f)** Means with various superscript letters vary significantly at *p* < 0.0001. MK, milk; UR, urine; PS, pus; SP, sputum; OD, optical density; TQ, thymoquinone; MIC, minimum inhibitory concentration; NAGase, β-*N*-acetylglucosaminidase; NA, non-adherent; WA, weakly adherent; MA, moderately adherent; SA, strongly adherent.

There were significant differences in the established biofilm inhibition among various concentrations of TQ, NAGase, and their combination against 10 MDR *S. aureus* isolates (*p* < 0.0001). TQ/NAGase showed the most significant (*p* < 0.0001) reduction in established biofilm in nine treated isolates, when compared with the control untreated ones. TQ at 0.5× MIC showed the most significant (*p* < 0.0001) reduction in established biofilm in one isolate with code no. PS1, when compared with the control untreated ones (**Figure 7A**).

The combination of TQ/NAGase resulted in a reduction of adherence from strong to weak in all tested isolates, with the exception of one isolate with code no. PS1, which exhibited a shift to moderate adherence (**Figure 7B**).

### Gene regulation

**Figure 8:**
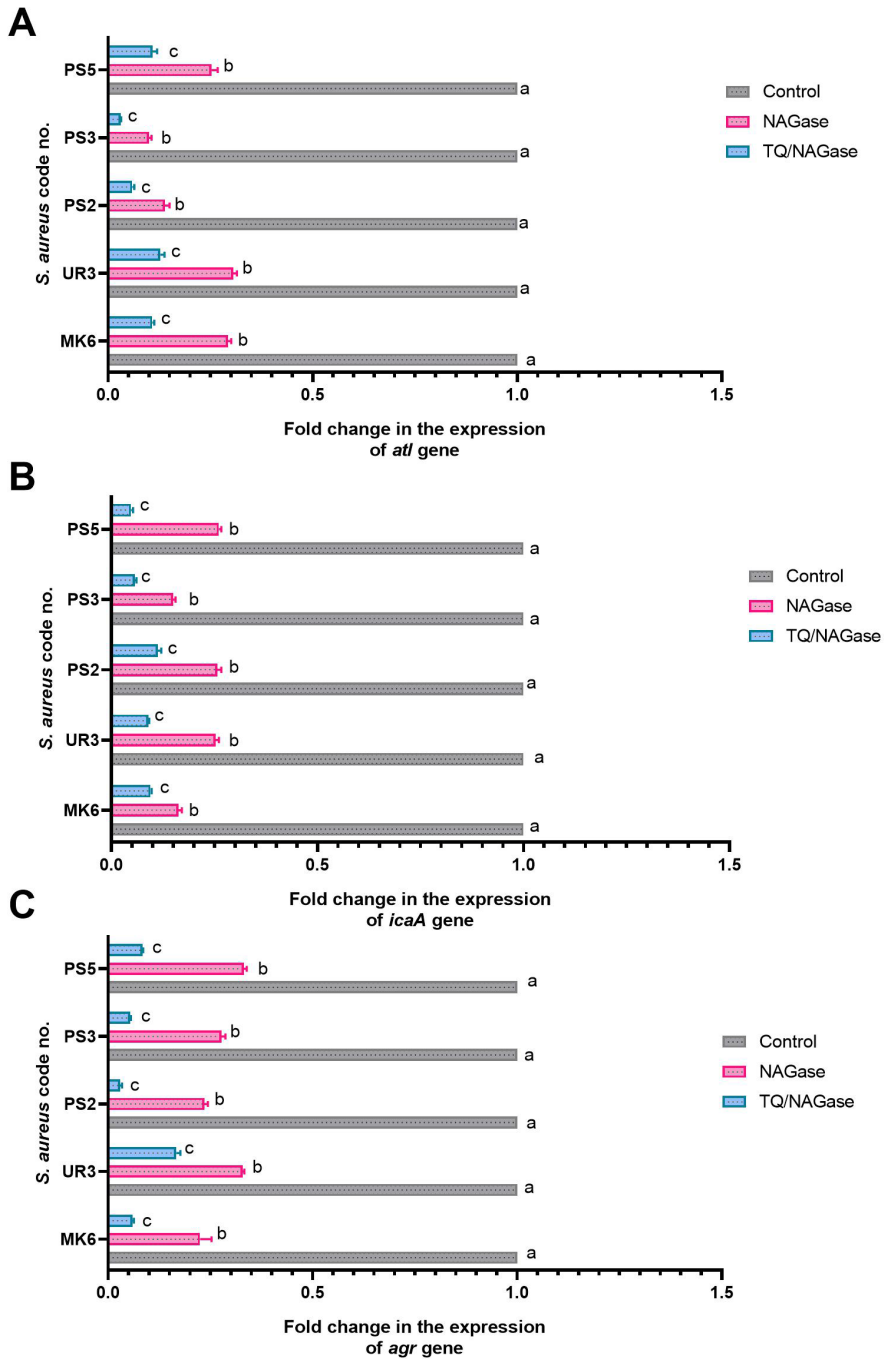
Fold changes in the expressions of *atl* **(A)**, *icaA* **(B)**, and *agr* **(C)** genes among *StaphylococcusS. aureus* isolates after treatment with NAGase, and NAGase, and thymoquinone combination (TQ/NAGase). Values represent the fold changes in comparison with the transcription levels of the control untreated isolates, which were assigned a value of 1. Results were expressed as the mean of three independent experiments ± standard error of the mean (SEM). ^a–c^ Means with various superscript letters vary significantly at *p* < 0.0001. NAGase, β-*N*-acetylglucosaminidase TQ, thymoquinone.

The qRT-PCR analysis of the transcription levels of *atl*, *icaA*, and *agr* genes in five strong biofilm-producing and MDR *S. aureus* isolates (code nos. MK6, UR3, PS2, PS3, and SP5) demonstrated various expression values when subjected to NAGase treatment alone compared to the combined treatment of TQ and NAGase, in relation to the untreated control group (**Figure 8**, **Supplementary Figure S1**). Of note, NAGase treatment resulted in a reduction of *atl* gene expression across all isolates, with the majority of fold changes falling below 0.5. The combination of TQ and NAGase further enhanced the suppression of the *atl* gene expression in *S. aureus* isolate code nos. PS2 and PS3, as indicated by the lower fold-change values. In all examined isolates, a consistent decrease in the expression of the *atl* gene was observed, although the extent of this drop varied slightly. The combination of TQ and NAGase typically led to a reduction in *icaA* gene expression; however, one isolate (code no. PS2) demonstrated a somewhat milder response to the combination treatment when compared to the effects of NAGase alone. Furthermore, both NAGase and TQ markedly diminished *agr* gene expression in all isolates when compared to the untreated controls. The combination treatment consistently resulted in a significant reduction in *agr* expression, exceeding the impact of NAGase used alone in the majority of analyzed isolates. The abovementioned results implied that TQ enhanced the inhibitory effect of NAGase on the expression of all analyzed genes, suggesting a possible synergistic relationship between the two compounds.

There were statistically significant differences (*p* < 0.0001) in the transcriptional modulation of *atl*, *icaA*, and *agr* genes among NAGase and TQ/NAGase-treated and control untreated *S. aureus* isolates. The most significant (*p* < 0.0001) downregulation of *atl*, *icaA*, and *agr* genes was recorded among TQ/NAGase-treated *S. aureus* isolates, followed by NAGase-treated *S. aureus* isolates, unlike the control untreated ones (**Figure 8**).

### Molecular docking analysis of thymoquinone’s impact on quorum sensing molecules

**Figure 9:**
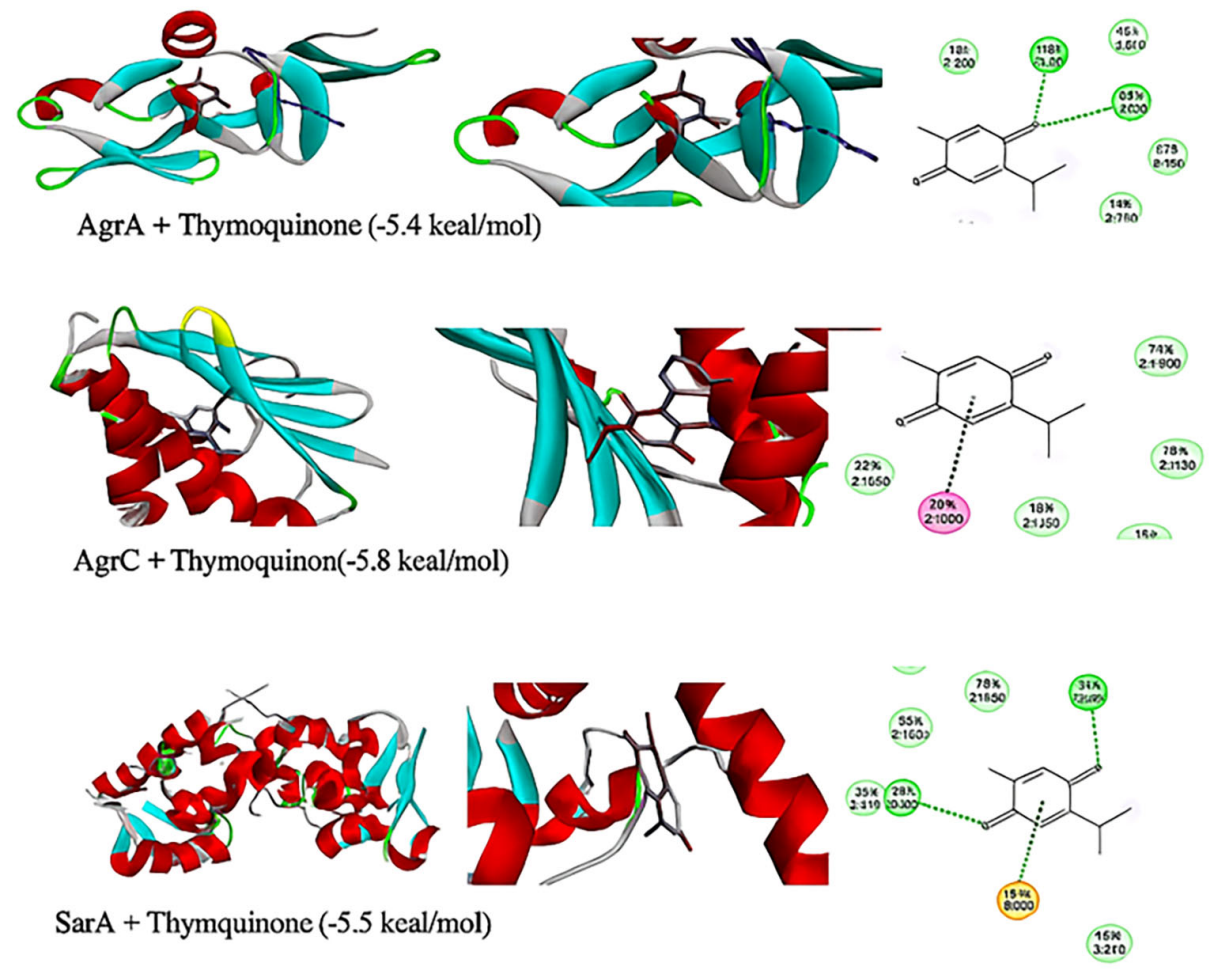
Three-dimensional representation and 2D model of thymoquinone ligand interactions with quorum sensing molecules (agrA, agrC, and sarA). Key colors of 2D representation: light green, Van der Waals; green, conventional hydrogen bonding; orange, Pi–anion; pink, Pi–Pi stacked.

**Figure 10:**
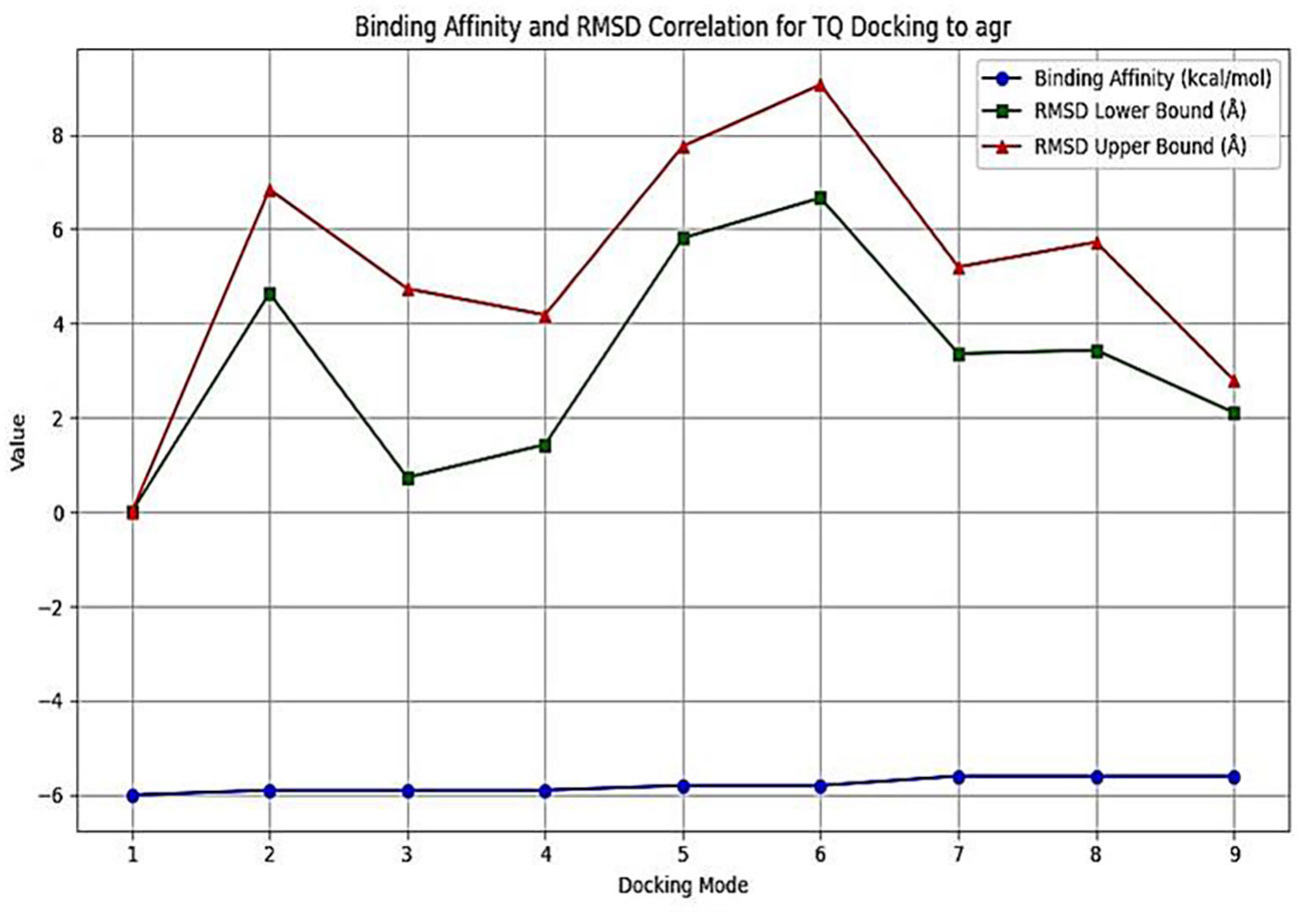
Binding affinity and RMSD for thymoquinone (TQ) docking to the agrA system. Binding affinity is represented by blue circles (left y-axis), while RMSD lower bounds are shown as green squares and upper bounds as red triangles (right y-axis) across nine docking configurations. The first configuration demonstrates the strongest binding affinity (−6.0 kcal/mol) and perfect pose stability (RMSD = 0.000), indicating an optimal interaction. Configurations 2–4 maintain similar affinities (−5.9 kcal/mol) with gradually increasing RMSD, suggesting minor structural deviations. Configurations 5–9 show reduced affinities (−5.8 to −5.6 kcal/mol) and higher RMSD values, indicating less favorable and more variable binding. Overall, lower RMSD values correlate with stronger binding, supporting the conclusion that TQ binds most effectively to agrA in a stable conformation. RMSD, root mean square deviation.

**Figure 11:**
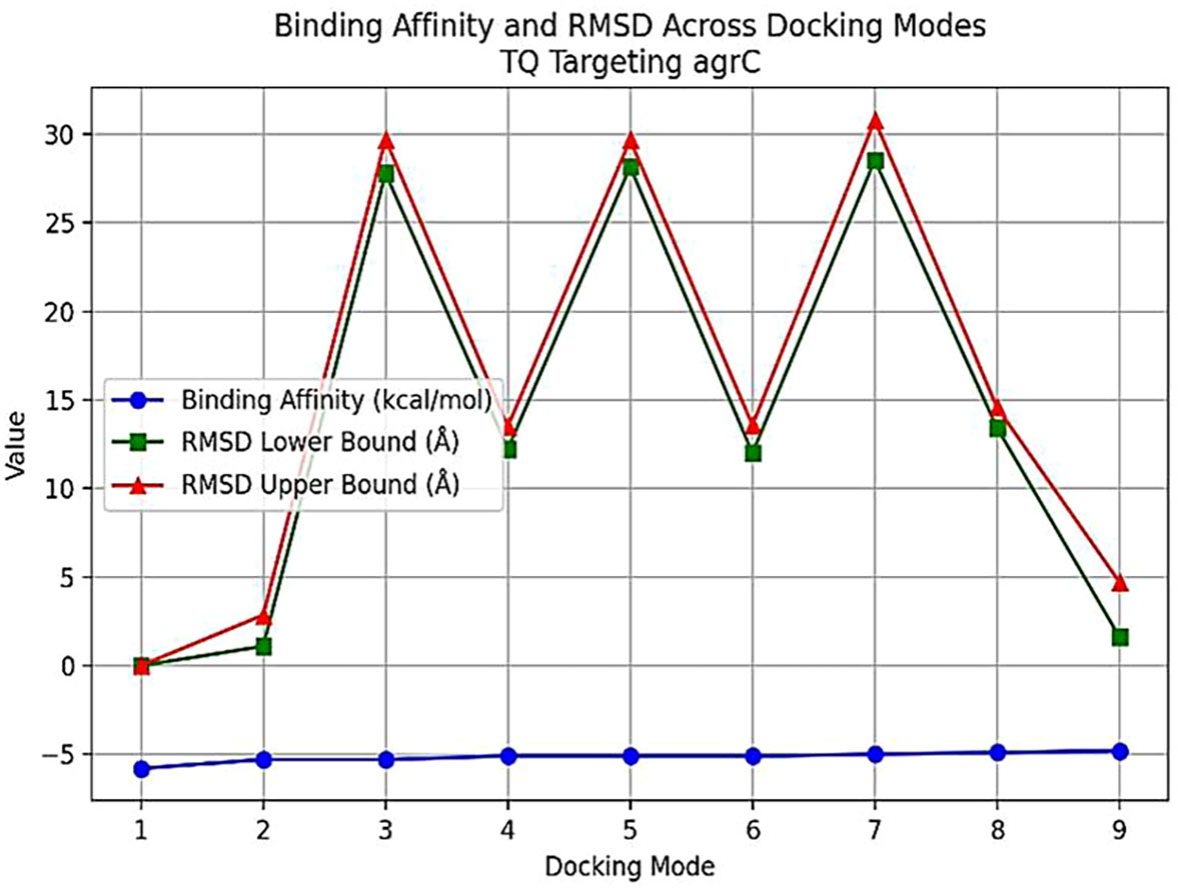
Binding affinity and RMSD across docking modes for TQ targeting agrC. The plot illustrates binding affinity (blue circles) and RMSD lower (green squares) and upper bounds (red triangles) for nine docking modes of thymoquinone (TQ) with agrC. The x-axis shows docking modes, while the y-axis displays binding affinity (kcal/mol) and RMSD **(A)**. Mode 1 demonstrates the strongest binding and lowest RMSD, indicating a highly stable pose. Higher modes show increased RMSD and reduced binding affinity, reflecting less favorable and more variable binding. RMSD, root mean square deviation.

**Figure 12:**
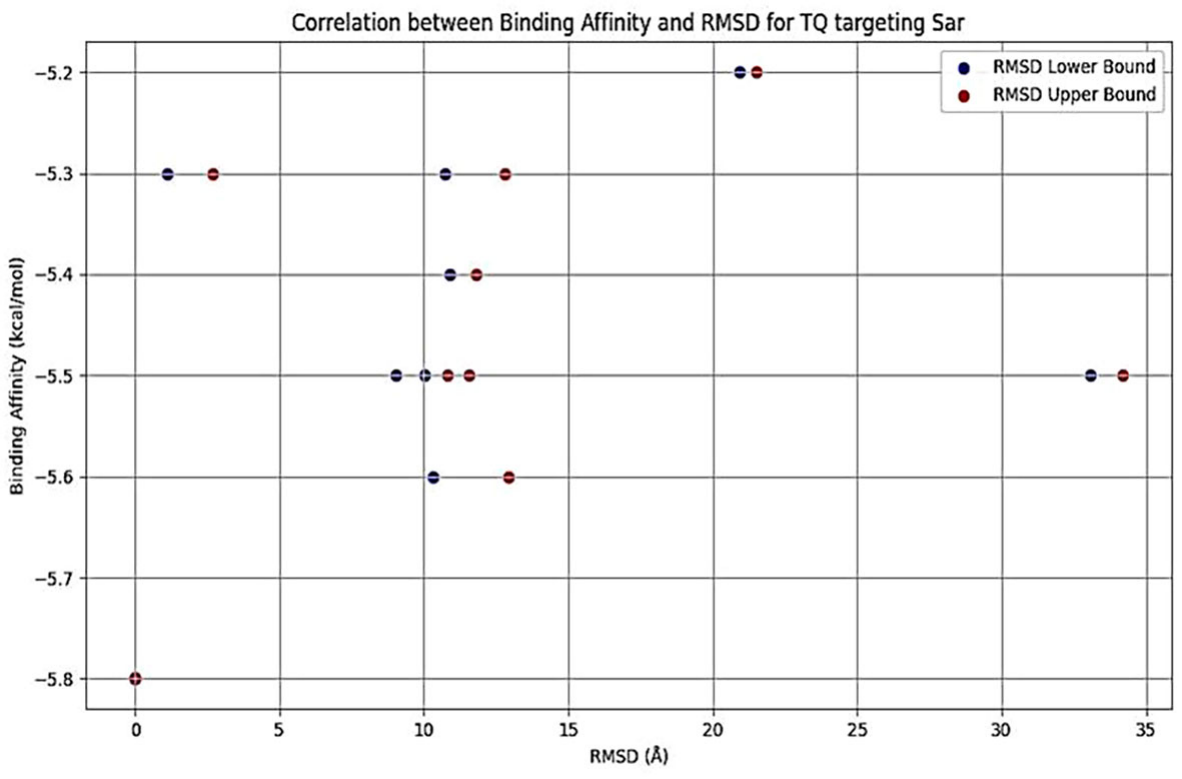
Binding affinity (blue dots) and RMSD lower (blue) and upper bounds (red) for different docking configurations of TQ with quorum sensing (sarA). As RMSD increases, binding affinity tends to decrease slightly, suggesting less optimal docking configurations. A wider range of RMSD values indicates greater deviation from the best pose, which may reflect less reliable binding. RMSD, root mean square deviation; TQ, thymoquinone.

Thymoquinone ligand interactions with QS sensing molecule, agrA, revealed that the TQ ligand formed conventional hydrogen bonds with two amino acid residues, HIS (A:169) and ARG (A:170) (**Figure 9**), suggesting a strong interaction between the ligand and the macromolecule. The docking results indicated that the ligand forms four Van der Waals interactions with the macromolecule, contributing to the overall binding affinity and stability of the protein–ligand. Thymoquinone ligand interactions with agrC-QS revealed the absence of conventional hydrogen bonding, suggesting that the ligand did not form strong electrostatic interactions with the macromolecule. Four Van der Waals interactions contributing to the ligand’s overall binding affinity to the macromolecule were noticed. An apparent Pi–Pi stacking interaction with PHE (A:382), which is important for protein–ligand binding, was observed, providing additional binding energy. Thymoquinone ligand interactions with the sarA-QS molecule revealed stability and specificity through two conventional hydrogen bonds with two amino acid residues, ASN (B:161) and TYR (B:162), and four van der Waals interactions in PHE (B:134), THR (A:117), VAL (A:116), and GLN (B:166). Pi–anion, indicative of attractive force, was observed in ASP (A:120). Analyses of root mean square deviation and binding affinity values obtained from docking simulations of thymoquinone with *S. aureus* regulator proteins agrA, agrC, and sarA, as part of MD simulations, were performed to provide preliminary insights into ligand binding stability. The data reveal that agrA exhibits the most favorable binding profile, with the strongest affinity (−6.0 kcal/mol) and perfect pose stability (RMSD = 0.0) in its top-ranked configuration (**Figure 10**, **Supplementary Table S3**). Subsequent configurations for agrA maintain relatively strong affinities (−5.9 to −5.6 kcal/mol) with moderate RMSD values, indicating stable and consistent binding. In contrast, agrC and sarA show weaker binding affinities (−5.8 to −4.8 kcal/mol for agrC; −5.8 to −5.2 kcal/mol for sarA) and significantly higher RMSD values in several configurations, particularly modes 3, 5, and 7 for agrC and mode 5 for sarA, suggesting unstable or less favorable binding poses (**Figures 11**, **12**, **Supplementary Table S3**). Notably, agrC exhibits extreme RMSD values exceeding 28 Å in some configurations, which may reflect poor docking accuracy or conformational mismatch. Overall, the data support the conclusion that TQ binds most effectively and stably to agrA, making it a promising target for further antimicrobial development. The correlation between lower RMSD and stronger binding affinity is consistently observed across all targets, reinforcing the importance of pose stability in docking-based drug design.

## Discussion

Bacterial biofilms pose a significant challenge to the elimination of bacteria from tissues due to their ubiquitous presence and resistance to antimicrobials. A promising strategy to combat biofilms involves using enzymes that break down the structural components of the biofilm matrix, successfully dispersing mature biofilms. Furthermore, enzymes that degrade polysaccharides can weaken biofilms, making embedded bacteria more susceptible to antimicrobials and thereby enhancing bacterial elimination (Jadhav et al., 2020; Abdelkader et al., 2023). Certain herbal compounds, particularly those containing safe enzymes that break down biofilms, exhibit a dual action that makes them extremely effective, as they not only kill bacteria but also disperse biofilms. Potent combinations of herbal active compounds, especially enzymes, are preferred alternatives to other conventional biofilm inhibitors, including peptide antibiotics, chelating agents, lantibiotics, and synthetic antibiofilm compounds. Thymoquinone, the active compound of *N. sativa*, is one of the most attractive antibacterial and antibiofilm agents against *S. aureus*, which can reduce the use of conventional antibiotics (Chaieb et al., 2011). This investigation focused on *S. aureus* because of its susceptibility to TQ, as supported by electron microscopy findings demonstrating its disruptive effects on the cell envelopes of Gram-positive bacteria (Dera et al., 2021; Wang et al., 2021) and fungi (Almshawit and Macreadie, 2017). This study investigated two plant-derived compounds for their antibiofilm properties. The first, NAGase from jack beans, was tested for its ability to prevent biofilm formation and break down existing biofilms. The second, TQ extracted from *N. sativa* seeds, was evaluated at various concentrations for its ability to inhibit biofilm formation and disperse the established biofilms, building on its known antibacterial properties. The mechanism by which TQ inhibits biofilm formation has not been previously studied. To enhance its effectiveness in preventing biofilm initiation and disrupting existing biofilms, a combination of NAGase and TQ (2.5 U/mL/1× MIC) was tested. Additionally, the molecular mechanism of TQ’s antibiofilm activity was investigated using computer simulations (*in silico* docking) and a real-time PCR assay.

The results demonstrated that NAGase exhibited notable inhibition of biofilm formation, detachment of the existing biofilm, and bactericidal activity against clinical *S. aureus* isolates in biofilms. This study reported that NAGase effectively dissolved biofilms and eradicated 40.9%–65.6% of existing biofilm at a concentration of 2.5 U/mL. Notably, this enzyme level falls within normal ranges in healthy individuals: 0.5–3.5 mU/mL in serum and 0.2–2.5 mU/mL in urine (Burtis et al., 2018). However, elevated levels up to 10 and 20 mU/mL have been reported in patients with chronic kidney disease and diabetic nephropathy, respectively, according to the Kidney Disease Outcomes Quality Initiative, which was updated and expanded into the Kidney Disease: Improving Global Outcomes guidelines (Levey et al., 2002). In healthy dogs, serum NAGase levels ranged from 0.02 to 3.63 U/L (Tassini et al., 2018). Additionally, research by Kausar et al. (2023) found that NAGase is linked to udder tissue damage caused by harmful bacteria. Notably, NAGase levels surged to 56.07 U/mL when somatic cell counts elevated to 178,645.83/mL. Moreover, this study found that TQ exhibited bactericidal activity against *S. aureus* with MIC values ranging from 2 to 100 µg/mL. Notably, TQ demonstrated robust ability to prevent biofilm formation and disrupt existing biofilms. Previous studies have also reported TQ bactericidal effects against *S. aureus* with MIC values ranging from 2 to 320 (Chaieb et al., 2011), 5 to 320 (Ahmad et al., 2024), 3 to 125 (Goel and Mishra, 2018), and 2 mg/mL (Al-Khalifa et al., 2021). In the present work, TQ reduced biofilm formation by 40.5%–57.9% at 0.5× MIC, 51.2%–77.4% at 1× MIC, and 59.4%–83.9% at 2× MIC after 24 h of treatment. These results are consistent with previous inhibitory values of 39%–54%, 48%–68%, and 61%–81% of cultivated *S. aureus* biofilm treated with 0.5× MIC, 1× MIC, and 2× MIC of TQ, respectively (Dera et al., 2021).

Herein, the plant-based combination of NAGase and TQ exhibited dual benefits, inhibiting and dispersing biofilms, with better scores after this combination. In contrast, a conventional approach requires a combination of the antibiotic ciprofloxacin with permethylated-β-CD, which enhances drug bioavailability to achieve similar effects (Abdelkader et al., 2023). Enzymes that degrade biofilms have gained widespread acceptance and are used in various therapeutic applications, including clinical practices. This study explored a safe, plant-based source of NAGase for breaking down biofilms, and it has been shown to prevent biofilm formation and increase the recovery of bacterial colonies from *S. aureus* biofilms. Dispersin B, an enzyme from the bacterium *Aggregatibacter actinomycetemcomitans*, is effective in breaking down PNAG, a key component of biofilms (Itoh et al., 2005). Dispersin B is being researched as a potential treatment for chronic wounds and antibiofilm therapy, with possible applications in medicine, cleaning products, coatings, and wound care (Kaplan, 2009). However, it may cause adverse reactions, including allergic responses, inflammation, gastrointestinal issues, and rare but severe side effects like anaphylaxis and blood-related complications. Further research is necessary before Dispersin B can be widely used in a medical practice. While serratiopeptidase is a potent antibiofilm agent with proteolytic activity, its use can cause temporary gastrointestinal side effects. Moreover, there is limited scientific evidence supporting its clinical applications (Jadhav et al., 2020). The molecular docking analysis of TQ with quorum sensing regulators agrA, agrC, and sarA reveals distinct binding profiles and pose stabilities. Notably, RMSD results provided more accurate insights than docking scores, with significant findings observed only for agrA. TQ formed strong interactions with agrA, including conventional hydrogen bonds with HIS (A:169) and ARG (A:170), and four Van der Waals contacts, indicating stable and specific binding. RMSD analysis showed perfect pose stability (RMSD = 0.0 Å) in the top-ranked configuration and moderate RMSD values in subsequent modes, supporting consistent binding. In contrast, agrC and sarA exhibited weaker affinities and higher RMSD values, with agrC showing extreme deviations exceeding 28 Å in some modes, suggesting poor docking accuracy or conformational mismatch. These findings underscore the importance of RMSD in evaluating docking reliability and highlight agrA as the most promising target for further antimicrobial development (Trott and Olson, 2010). It was demonstrated that these glycoside hydrolases selectively target and degrade the exopolysaccharide component of the biofilm matrix (Baker et al., 2016). The synthesis of PIA is inhibited due to the reduced expression of *ica*, *agr*, and *atl* genes (Novick et al., 1993; Cramton et al., 1999; Xiang et al., 2020). This decrease leads to lower QS signal production, which, in turn, impairs cell separation and biofilm formation. In the present work, the treatment of *S. aureus* isolates with TQ/NAGase resulted in substantial reductions in the expression of *atl*, *agr*, and *ica* genes. This decrease in gene expression was accompanied by a significant dispersion of biofilms in microplates by 61.8%–73.8% compared to untreated controls. Additional studies are needed to determine the optimal dosage and administration method of NAGase, including topical application and injection. Specifically, intramammary infusion requires further investigation, as NAGase levels were higher (56.07 U/mL) in cases of mastitis (Kausar et al., 2023) compared to the level (2.5 U/mL) used in this study. A promising treatment approach could involve using a combination of NAGase and TQ antibiofilm agents based on the following reasons. First, traditional antibiofilm agents, which use enzymes to break down PIA or proteins, have limited use in medicine. In contrast, NAGase, a naturally occurring enzyme found in cellular lysosomes, appears to be a safer and more effective alternative. It successfully disperses biofilms at levels lower than those typically found in healthy individuals and those associated with diseases. Second, the TQ/NAGase combination resulted in a synergistic reduction of the existing biofilm by 61.8%–73.8%, which can be attributed to the presence of two antibiofilm components: one targeting QS and the other targeting PIA degradation. Third, the combination did not need additional antibiotics because TQ exhibited the necessary antibacterial properties to combat *S. aureus* extracted from the treated biofilm. Finally, they both interrupted cell communication by suppressing the production of QS genes and their expressed molecules.

## Conclusions

β-*N*-acetylglucosaminidase and TQ represent promising plant-based compounds that can be utilized safely as adjunct agents alongside antibacterial treatments and biofilm disruptors in the management of *S. aureus*. NAGase has been proven to be a potent biofilm dispersant enzyme, effectively degrading extracellular polysaccharides and thereby preventing the formation of biofilms. Moreover, combining TQ with NAGase yielded even more impressive outcomes. This synergistic effect led to a significant reduction of existing biofilm through three distinct mechanisms: i) the enzymatic dispersion of PIA; ii) the inhibition of QS signaling pathways via the downregulation of *agr*-QS gene and the suppression of their agrA, agrC, and sarA-QS sensing molecules; and iii) the bactericidal action against clinical *S. aureus* isolates.

## Data Availability

The datasets presented in this study can be found in online repositories. The names of the repository/repositories and accession number(s) can be found in the article/**Supplementary Material**.
