# Supplementary material for: Thymoquinone/β-N-acetylglucosaminidase, a novel plant-derived combination, inhibited quorum sensing signaling pathways and disrupted biofilm in Staphylococcus aureus
Source: Front Cell Infect Microbiol. 2025 Oct 23;15:1686764. doi: 10.3389/fcimb.2025.1686764 (PMC12589069; doi:10.3389/fcimb.2025.1686764)
Supplement: Supplementary file 1 [file DataSheet1.pdf]

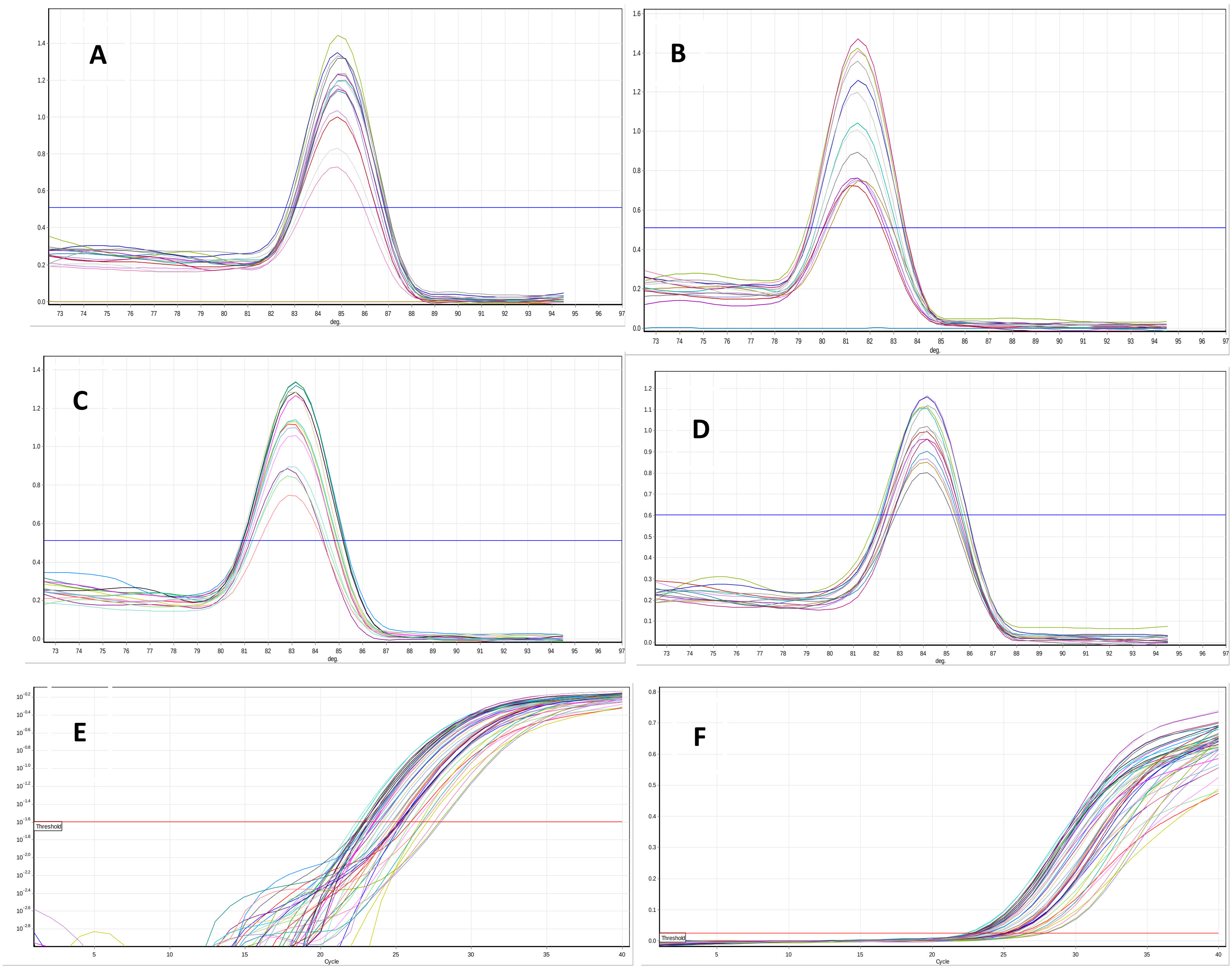

Figure S1: Quantitative real-time PCR showing melting curves of *16sRNA* (A), *agr*(B), *ica*(C) *and atl* (D)genes and amplification plot (log) (E) and (linear)(F).
