# Supplementary material for: Thymoquinone/β-N-acetylglucosaminidase, a novel plant-derived combination, inhibited quorum sensing signaling pathways and disrupted biofilm in Staphylococcus aureus
Source: Front Cell Infect Microbiol. 2025 Oct 23;15:1686764. doi: 10.3389/fcimb.2025.1686764 (PMC12589069; doi:10.3389/fcimb.2025.1686764)
Supplement: Supplementary file 2 [file Table1.docx]

**Table S1.** Antimicrobial susceptibility patterns of *S. aureus* isolates recovered from mastitis milk samples

| **Isolate**  **code No.** | **Resistance pattern to antimicrobial agents** | | | | | | | | | **No. of drugs to which the isolates were resistant** |
| --- | --- | --- | --- | --- | --- | --- | --- | --- | --- | --- |
|  | **OX, DA, FF** | **AMC** | **AZM** | **SXT** | **CIP** | **DO** | **CN** | **C** | **LNZ** |  |
| **MK1** | R | R | R | R | R | R | R | R |  | 10 |
| **MK2** | R | R | R | R | R | R | R |  |  | 9 |
| **MK3** | R | R | R |  | R | R |  | R |  | 8 |
| **MK4** | R | R | R |  |  |  | R |  | R | 7 |
| **MK5** | R | R | R | R |  |  | R |  |  | 7 |
| **MK6** | R | R | R | R | R |  |  |  |  | 7 |
| **MK7** | R | R | R |  | R |  |  |  |  | 6 |
| **MK8** | R | R | R | R |  |  |  |  |  | 6 |
| **MK9** | R |  | R |  | R |  |  |  |  | 5 |
| **MK10** | R | R |  |  |  |  |  | R |  | 5 |
| **MK11** | R | R |  |  |  | R |  |  |  | 5 |
| **MK13** | R | R |  | R |  |  |  |  |  | 5 |
| **MK12, MK14-20** | R | R | R |  |  |  |  |  |  | 5 |
| **MK21** | R |  | R |  |  |  |  |  |  | 4 |
| **MK22-24** | R | R |  |  |  |  |  |  |  | 4 |
| **MK25** | R |  |  |  |  |  |  |  |  | 3 |
| **Resistance percentage** | 100 | 88 | 72 | 24 | 24 | 16 | 16 | 12 | 4 |  |

MK: milk, R: resistant, OX: oxacillin, DA: clindamycin, FF: fosfomycin, AMC: amoxicillinclavulanic acid, AZM: azithromycin, SXT: trimethoprimsulfamethaxole, CIP: ciprofloxacin, DO: doxycycline, CN: gentamycin, C: chloramphenicol, LNZ: linezolid.

**Table S2.** Antimicrobial susceptibility patterns of *S. aureus* isolates recovered from human sources

| **Isolate**  **code No.** | **Resistance pattern to antimicrobial agents** | | | | | | | | **No. of drugs**  **to which the isolates were resistant** |
| --- | --- | --- | --- | --- | --- | --- | --- | --- | --- |
|  | **OX, DA**  **FF, AMC** | **AZM** | **CN** | **SXT** | **CIP** | **C** | **DO** | **LNZ** |  |
| **PS1** | R | R | R | R | R |  |  | R | 9 |
| **PS2** | R | R | R | R | R | R |  |  | 9 |
| **PS3** | R | R | R | R |  |  | R |  | 8 |
| **PS4** | R | R |  | R | R |  |  |  | 7 |
| **SP5** | R | R | R |  | R |  |  |  | 7 |
| **PS7-8** | R |  |  |  |  |  |  |  | 4 |
| **UR1** | R | R | R | R | R |  |  |  | 8 |
| **SP6, UR2** | R | R | R | R |  |  |  |  | 7 |
| **UR3** | R | R | R |  |  |  |  |  | 6 |
| **Resistance percentage** | 100 | 81.8 | 72.7 | 63.6 | 45.5 | 9.1 | 9.1 | 9.1 |  |

PS: pus, SP: sputum, UR: urine, R: resistant, OX: oxacillin DA: clindamycin, FF: fosfomycin, AMC: amoxicillinclavulanic acid, AZM: azithromycin, CN: gentamycin, SXT: trimethoprimsulfamethaxole, CIP: ciprofloxacin, C: chloramphenicol, DO: doxycycline, LNZ: linezolid.

# **Table S3.** Correlation between binding affinity and root mean square deviation for thymoquinone docking with *S. aureus* regulators agrA, agrC and sarA

| **Target** | **Mode** | **Affinity (kcal/**  **mol)** | **RMSD**  **Lower Bound** | **RMSD Upper Bound** |
| --- | --- | --- | --- | --- |
| **agrA** | 1 | -6.0 | 0.0 | 0.0 |
|  | 2 | -5.9 | 4.639 | 6.842 |
|  | 3 | -5.9 | 0.73 | 4.735 |
|  | 4 | -5.9 | 1.43 | 4.179 |
|  | 5 | -5.8 | 5.807 | 7.755 |
|  | 6 | -5.8 | 6.66 | 9.061 |
|  | 7 | -5.6 | 3.358 | 5.195 |
|  | 8 | -5.6 | 3.432 | 5.723 |
|  | 9 | -5.6 | 2.108 | 2.795 |
| **agrC** | 1 | -5.8 | 0.0 | 0.0 |
|  | 2 | -5.3 | 1.097 | 2.856 |
|  | 3 | -5.3 | 27.786 | 29.706 |
|  | 4 | -5.1 | 12.237 | 13.5 |
|  | 5 | -5.1 | 28.146 | 29.662 |
|  | 6 | -5.1 | 12.004 | 13.579 |
|  | 7 | -5.0 | 28.521 | 30.78 |
|  | 8 | -4.9 | 13.421 | 14.571 |
|  | 9 | -4.8 | 1.62 | 4.722 |
| **sarA** | 1 | -5.8 | 0.0 | 0.0 |
|  | 2 | -5.6 | 10.327 | 12.912 |
|  | 3 | -5.5 | 9.012 | 10.825 |
|  | 4 | -5.5 | 10.044 | 11.561 |
|  | 5 | -5.5 | 33.079 | 34.176 |
|  | 6 | -5.4 | 10.893 | 11.797 |
|  | 7 | -5.3 | 10.71 | 12.815 |
|  | 8 | -5.3 | 1.11 | 2.706 |
|  | 9 | -5.2 | 20.933 | 21.509 |

**RMSD:** root mean square deviation
